# WTAP‐Mediated m^6^A Modification Targets the LRP1‐Lipid Metabolism Axis to Regulate Joint Cartilage Regeneration

**DOI:** 10.1002/advs.75479

**Published:** 2026-05-06

**Authors:** Chenyan Huang, Chenyu Deng, Zhengrong Gao, Chen Chen, Huimin Zheng, Yue Yang, Yan Wei

**Affiliations:** ^1^ Department of Geriatric Dentistry NMPA Key Laboratory for Dental Materials Peking University School and Hospital of Stomatology & National Center of Stomatology & National Clinical Research Center for Oral Diseases & National Engineering Research Center of Oral Biomaterials and Digital Medical Devices Beijing P. R. China; ^2^ Peking University Hospital of Stomatology Sanya Division (Sanya Stomatology Center) Sanya Hainan P. R. China; ^3^ Nanjing Stomatological Hospital Affiliated Hospital of Medical School Institute of Stomatology Nanjing University Nanjing P. R. China; ^4^ Department of Orthodontics Cranial‐Facial Growth and Development Center Peking University School and Hospital of Stomatology Beijing P. R. China; ^5^ Department of Prosthodontics The First Clinical Division Peking University School and Hospital of Stomatology Beijing P. R. China

**Keywords:** cartilage regeneration, drugs screening, lipid metabolism, low‐density lipoprotein receptor‐related protein 1 (LRP1), wilms' tumor 1‐associating protein (WTAP)

## Abstract

Osteoarthritis (OA) arises from impaired epigenetic coordination of inflammatory and metabolic cues, leading to compromised cartilage homeostasis. Such coordination is partly governed by ribonucleic acid (RNA) epigenetic mechanisms, however, the role of the predominant RNA modification N^6^‐methyladenosine (m^6^A) in this process remains unclear. Herein, we identify an epigenetic–metabolic pathway in which Wilms' Tumor 1‐Associating Protein (WTAP)‐mediated m^6^A modification stabilizes low‐density lipoprotein receptor‐related protein 1 (LRP1) and redirects lipid metabolism toward chondrogenesis. Loss‐of‐function assays demonstrate that WTAP is required for the chondrogenic differentiation of BMSCs, as its depletion suppresses the expression of multiple cartilage‐associated genes. Mechanistically, WTAP enhances m^6^A methylation and stabilizes *Lrp1* transcripts, a key regulator of cholesterol metabolism and matrix synthesis, thereby driving lipid metabolic reprogramming toward chondrogenesis. Structure‐based screening identified silibinin and estradiol benzoate as LRP1‐specific agonists that activate the WTAP–LRP1 pathway to promote cartilage repair in vivo. Collectively, our findings establish m^6^A‐dependent metabolic reprogramming as a pivotal epigenetic mechanism of cartilage regeneration with therapeutic potential for promoting chondrogenesis.

## Graphical abstract

WTAP drives cartilage regeneration by activating an LRP1‐dependent lipid metabolic program in macrophages, enhancing IL‐10 and TGF‐β secretion to promote chondrogenic differentiation. Leveraging this mechanism, virtual screening identifies LRP1‐targeting compounds that effectively stimulate cartilage repair, highlighting a druggable epigenetic–metabolic axis for osteoarthritis therapy.

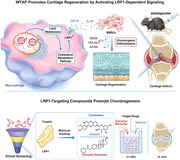

## 1 Introduction

With the accelerating global aging population and the growing prevalence of obesity, the incidence of osteoarthritis (OA) continues to rise [1, 2]. The primary pathological hallmark of OA is progressive degeneration of articular cartilage [3, 4, 5].

As a specialized connective tissue covering articular surfaces, articular cartilage plays a critical role in reducing friction and dissipating mechanical loads, thereby maintaining joint function and quality of life [6, 7]. However, owing to the absence of blood vessels, nerves, and lymphatic vessels, articular cartilage exhibits a markedly limited capacity for self‐repair [8, 9]. Therefore, the development of minimally invasive and efficient targeted therapies for cartilage regeneration in OA is of substantial clinical importance. At the molecular level, cartilage regeneration depends on precise regulation of gene expression, including epigenetic mechanisms that coordinate cellular differentiation and tissue repair.

N6‐methyladenosine (m^6^A) modification is one of the most prevalent epigenetic ribonucleic acid (RNA) modifications and plays essential roles in tissue development, disease progression, and regeneration [10]. Accumulating evidence indicates that m^6^A regulates key signaling pathways involved in chondrocyte proliferation, migration, and differentiation, thereby contributing to cartilage repair and regeneration [11]. Methyltransferase‐like 3 (METTL3)‐mediated m^6^A modification has been shown to regulate the chondrogenic differentiation of synovial mesenchymal stem cells (SMSCs) through the dentin matrix acidic phosphoprotein 1–YTH domain‐containing protein 1 signaling axis, thereby modulating cartilage‐specific genes such as SRY‐box transcription factor 9 (*Sox9), Aggrecan (Acan)*, and Collagen type II alpha 1 chain (*Col2α1)* [12, 13]. Beyond regulating lineage‐specific transcriptional programs, m^6^A has also emerged as a key regulator of cellular metabolic activity [14], controlling lipid homeostasis [15], cholesterol handling [16], and mitochondrial function [17] in multiple tissues. Given that metabolic reprogramming is increasingly recognized as an essential determinant of chondrocyte differentiation [18], m^6^A‐dependent metabolic regulation may represent a critical layer of control in cartilage regeneration. Wilms' tumor 1‐associating protein (WTAP), a core subunit of the m^6^A methyltransferase complex METTL3/14, is responsible for substrate recruitment and the proper localization of this complex [19, 20]. The knockdown of *wtap* in zebrafish embryos results in tissue differentiation defects and increased apoptosis [20], indicating WTAP's critical role in the epitranscriptomic regulation of RNA metabolism. Our preliminary studies further demonstrate that WTAP‐mediated m^6^A modification regulates bone tissue repair by modulating macrophage polarization [21]. However, how WTAP‐mediated m^6^A modification integrates metabolic pathways to influence chondrogenesis remains unknown.

Metabolic regulation is central to maintain immune microenvironment homeostasis and governing cell differentiation, with lipid metabolism playing a particularly important role in the onset and progression of OA. Previous studies have demonstrated that inhibition of excessive cholesterol synthesis in OA can mitigate cartilage degradation [22]. Members of the low‐density lipoprotein receptor‐related protein (LRP) family serve as pivotal receptors of lipid metabolism and are implicated in metabolic reprogramming during tissue regeneration. Wang et al. conducted sequential screening to identify ouabain and digoxin, confirming their capacity to enhance chondrocyte differentiation and further identifying LRP4 as the functional target of digoxin [23]. This finding underscores the direct involvement of the LRP family, as metabolism‐related receptors, in the regulation of cartilage homeostasis and regeneration. However, the role of WTAP‐mediated m^6^A modification in cartilage repair through lipid metabolic receptors, as well as its underlying molecular mechanisms, remains poorly understood, thereby limiting the development of targeted epigenetic therapies for OA.

**FIGURE 1:**
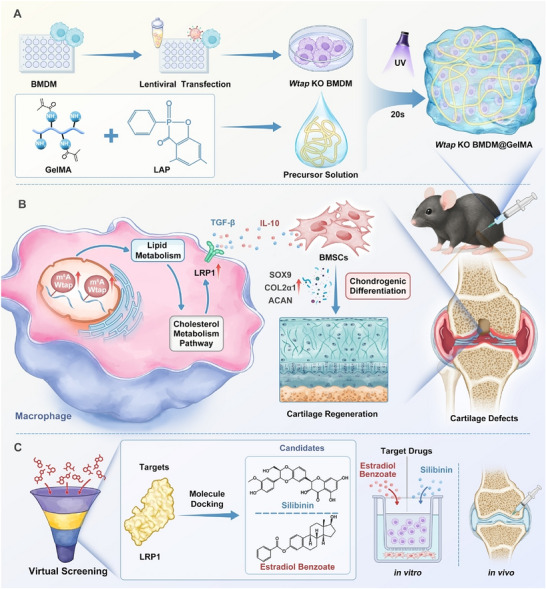
Design and mechanism of the WTAP‐mediated LRP1‐lipid metabolism axis in cartilage repair. (A) Schematic illustration of *Wtap* KO BMDM@GelMA preparation. (B) WTAP‐mediated LRP1 expression via lipid metabolism for cartilage regeneration. (C) Identification of silibinin and estradiol benzoate as LRP1‐targeting chondroprotective agents for cartilage repair.

In this study, we identified LRP1 as a pivotal lipid metabolic‐related receptor through integrated multi‐omics analyses and further delineate its role in WTAP‐mediated m^6^A–regulated chondrogenesis. Initially, utilizing macrophage‐specific *Wtap* knockout (*Wtap* KO) models in both in vitro and in vivo settings, we demonstrate that WTAP plays a promotive role in cartilage regeneration. Subsequently, integrated transcriptomic and metabolomic analyses demonstrated that LRP1‐mediated lipid metabolism represents the central mechanism underlying WTAP‐driven cartilage regeneration. Moreover, we performed LRP1‐targeted drug screening and identified small‐molecule compounds with high binding affinity to LRP1. Collectively, this study establishes a solid scientific foundation for novel targeted strategies to address OA‐associated cartilage damage (Figure 1).

## 2 Results

### 2.1 WTAP Facilitates Cartilage Regeneration In Vitro and In Vivo

**FIGURE 2:**
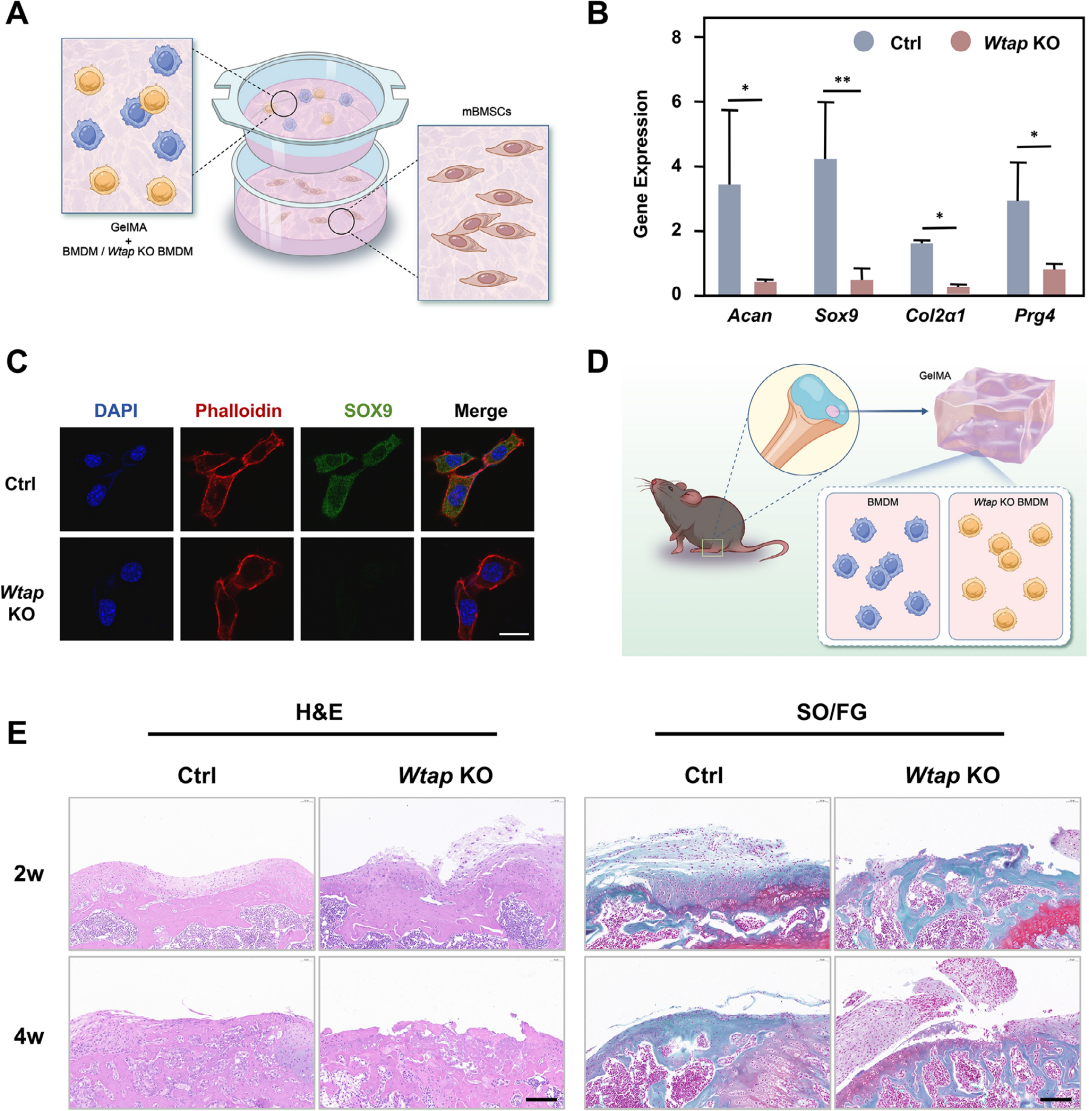
WTAP facilitates cartilage regeneration in vitro and in vivo. (A) Schematic of the co‐culture system of WT/*Wtap* KO BMDMs and mBMSCs. (B) RT‐qPCR analysis of chondrogenesis‐related genes (*Acan*, *Sox9*, *Col2α1*, and *Prg4*) in mBMSCs after co‑culture with WT/*Wtap* KO BMDMs. (C) Representative immunofluorescence images of SOX9 protein expression (green) in mBMSCs (scale bar: 25 µm). (D) Illustration of the implantation of WT/*Wtap* KO BMDM@GelMA into the mouse knee joint cartilage defect. (E) H&E and Safranin O‐Fast Green staining of mouse knee joint cartilage in the *Wtap* KO and control groups at 2 and 4 weeks (*n* = 5; scale bar: 100 µm). Data are presented as means ± SD. ^*^ *p* < 0.05, ^**^ *p* < 0.01.

M^6^A is a dynamic and reversible post‐transcriptional modification that extensively participates in regulating mRNA function. This modification exerts critical effects on various physiological processes, including organismal growth, substance and energy metabolism, and inflammatory responses [24]. To investigate the role of the WTAP‐mediated m^6^A modification in cartilage defect repair, we successfully generated *Wtap* KO bone marrow‐derived macrophages (BMDMs) via lentiviral‐mediated transfection (Figure S1). Wild‐type (WT) and *Wtap* KO BMDMs were co‐cultured with mouse bone marrow mesenchymal stem cells (mBMSCs), respectively (Figure 2A). Real‐time quantitative polymerase chain reaction (RT‑qPCR) results showed that the mRNA expression levels of chondrogenesis‐related genes in the *Wtap* KO group, such as *Acan, Sox9, Col2α1*, and Proteoglycan 4 (*Prg4)*, were significantly lower than those in the control group (Figure 2B). This finding suggests that *Wtap* knockout in BMDMs impairs the chondrogenic differentiation capacity of mBMSCs. Meanwhile, we examined the expression of chondrogenesis‐related proteins. Immunofluorescence assays revealed that the green fluorescence intensity of the SOX9 protein was markedly weaker in the *Wtap* KO group compared with the control group (Figure 2C). Therefore, *Wtap* knockout in BMDMs significantly downregulated the expression of both chondrogenesis‐related genes and proteins in mBMSCs, indicating that WTAP promotes the chondrogenic differentiation of mBMSCs.

We further implanted gelatin methacryloyl (GelMA) hydrogel‐coated *Wtap* WT/KO BMDMs into the cartilage defect sites of mouse knee joints (Figure 2D). Tissue samples from the knee joint defect healing areas were collected at 2 and 4 weeks after surgery. Hematoxylin and eosin (H&E) staining revealed a significant reduced cartilage defect area and greater coverage of newly formed bone tissue in the control group compared with the *Wtap* KO group. Furthermore, Safranin O‐Fast Green staining demonstrated a marked increase in the number of newly formed chondrocytes and superior defect healing in the control group relative to the *Wtap* KO group (Figure 2E). Notably, the volume of newly formed bone tissue was substantially greater at 4 weeks post‐implantation than at 2 weeks, indicating a time‐dependent enhancement of tissue repair and regenerative capacity. These histological results demonstrate that *Wtap* knockout in BMDMs significantly impairs articular cartilage injury repair, confirming a promote role of WTAP in cartilage regeneration. However, the underlying mechanism by which WTAP mediates the cartilage regenerative process remains unclear and requires further investigation.

### 2.2 Transcriptomic Analysis Reveals WTAP‐Dependent Upregulation of Chondrogenic Genes

**FIGURE 3:**
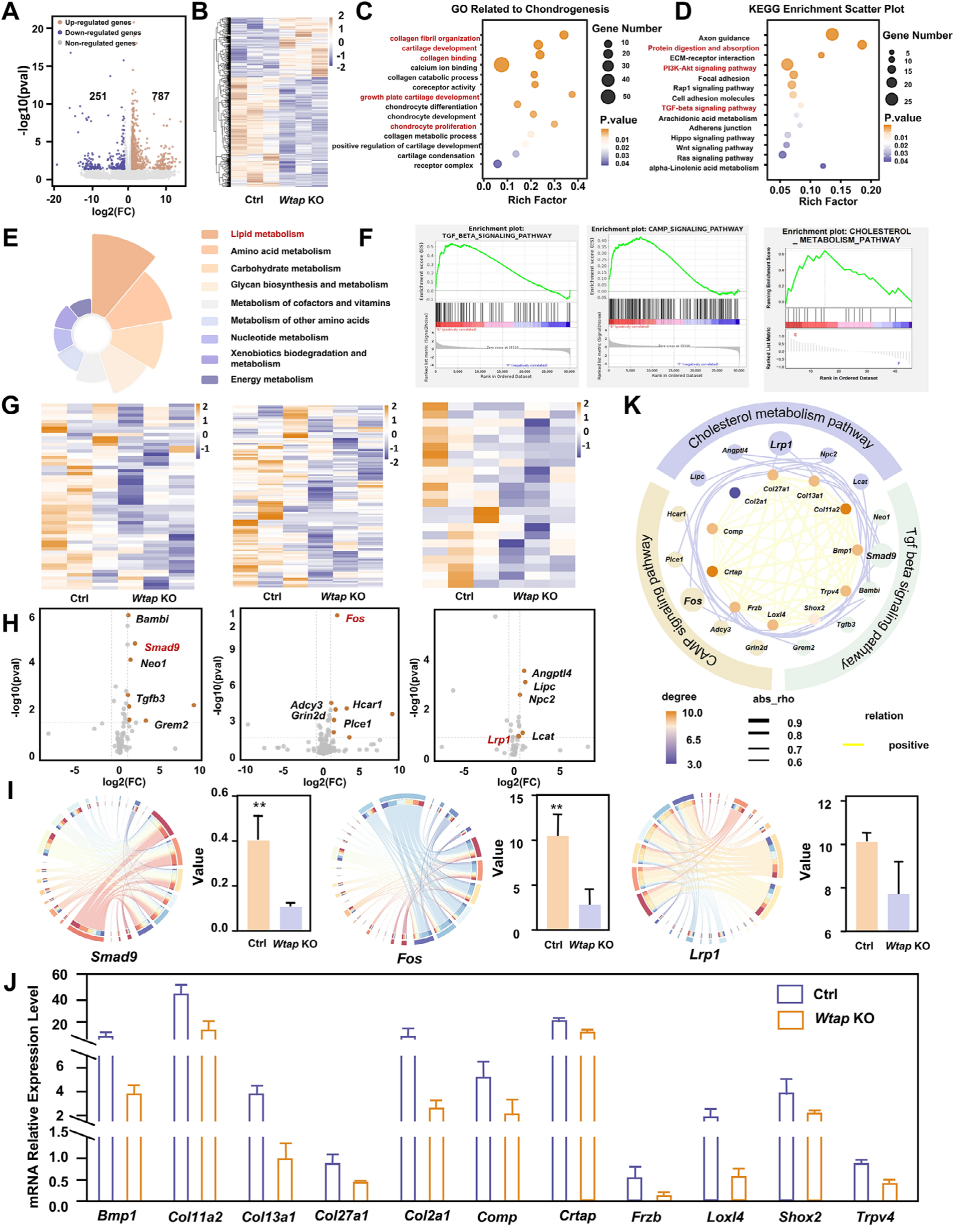
Transcriptome sequencing reveals WTAP‐induced upregulation of chondrogenic genes. (A) Volcano plot showing 787 upregulated and 251 downregulated DEGs between the *Wtap* KO and control groups. (B) Heatmap of selected DEGs between the two groups. (C) GO enrichment analysis of the DEGs. (D) KEGG enriched biological process and signaling pathway based on DEGs. (E) KEGG enrichment analysis of metabolism‐related DEGs following *Wtap* knockout. (F) GSEA of the TGF‐β signaling pathway, cAMP signaling pathway, and cholesterol metabolism after *Wtap* knockout. (G) Heatmap of DEGs in the three core pathways (from F) between the two groups. (H) Volcano plot of core genes from the three key pathways (from F) between the two groups. (I) Relative abundance and expression levels of *Smad9, Fos*, and *Lrp1* were identified as significantly altered genes in these pathways. (J) Relative expression analysis of chondrogenic differentiation‐related genes in the *Wtap* KO group compared with the control. (K) PPI network of chondrogenic genes and the identified signaling pathways. Data are presented as means ± SD. ^**^ *p* < 0.01.

To investigate the epigenetic mechanism by which WTAP regulates cartilage regeneration, we performed transcriptomic sequencing on the aforementioned knee joint cartilage defect healing tissues from the *Wtap* KO and control groups at 2 and 4 weeks. At 2 weeks, analysis identified 928 differentially expressed genes (DEGs), with 528 upregulated and 400 downregulated in control group relative to *Wtap* KO group (Figure S2A). As the tissue healing progressed to 4 weeks, the number of DEGs increased to 1038, including 787 upregulated and 251 downregulated in the control group compared with the *Wtap* KO group (Figure 3A). Clustering heatmap further confirmed distinct gene expression profiles between the two groups (Figure S2B; Figure 3B). In summary, these results demonstrate that WTAP induces substantial alterations in gene expression during the tissue healing process, highlighting its regulatory role in modulating transcriptional programs associated with cartilage regeneration.

Enrichment analysis of DEGs revealed significant enrichment in cartilage regeneration‐related biological processes. At 2 weeks, Gene Ontology (GO) enrichment terms included cartilage development and chondrocyte differentiation (Figure S2C). By 4 weeks, the enriched GO terms shifted toward collagen fibril organization, collagen binding, and chondrocyte proliferation (Figure 3C). Kyoto Encyclopedia of Genes and Genomes (KEGG) pathway analysis highlighted regeneration‐related pathways at 2 weeks, including phosphatidylinositol 3‐kinase‐protein kinase B (PI3K‐AKT), cyclic adenosine monophosphate (cAMP), and rat sarcoma (Ras) pathways (Figure S2D), while extracellular matrix (ECM)‐receptor interaction, transforming growth factor‑β (TGF‐β), Hippo, and Wnt signaling were enriched at 4 weeks (Figure 3D). These signaling pathways form an interactive network that regulates cell proliferation, differentiation, and migration. Additionally, DEGs were enriched in multiple metabolism‐related pathways, such as ether lipid metabolism, linoleic acid metabolism, and arachidonic acid metabolism (Figure S2D; Figure 3D). These pathways support crucial cellular functions, including energy production [25], synthesis of membrane components [26], and signaling molecules generation [27], suggesting that metabolic pathways play a critical role in articular cartilage regeneration. Further metabolic categorization confirmed lipid metabolism was the most significantly enriched among metabolic pathways at both stages (Figure S2E; Figure 3E). This result indicates that lipid metabolism plays a key regulatory role in WTAP‐mediated cartilage regeneration.

To identify key regulatory factors, Gene Set Enrichment Analysis (GSEA) was performed on the transcriptomic data. At 2 weeks, pathways including PI3K‐AKT signaling, Wnt signaling, and protein digestion and absorption were highly enriched (Figure S3A). This indicates significant activation of signaling pathways closely associated with cell proliferation and tissue regeneration during the early phase of cartilage repair. Heatmap and volcano plot visualizations showed a general downregulation of core genes in these pathways in the *Wtap* KO group (Figure S3B,C). To further elucidate the molecular processes underlying cartilage regeneration, we analyzed DEGs enriched within cartilage regeneration‐related pathways. Notably, cartilage‐related genes (e.g., *Sox9, Col2α1, Acan, Col11α2*, and *Col27α1*) were also found to be significantly downregulated in the *Wtap* KO (Figure S3D). At 4 weeks, the highly enriched processes shifted to TGF‐β signaling, cAMP signaling, and cholesterol metabolism (Figure 3F), suggesting robust activation of pathways linked to tissue regeneration and lipid metabolism during the late stage of cartilage repair. To visualize these GSEA results, we generated pathway‐specific gene set profiles. Both the heatmap and the volcano plot displayed a downregulation trend of key genes in the *Wtap* KO group compared with the control (Figure 3G,H). This transcriptional downregulation may impede stem cell chondrogenic differentiation by disrupting the aforementioned signaling cascades. DEG analysis further identified mothers against decapentaplegic homolog 9 (*Smad9), FBJ murine osteosarcoma viral oncogene homolog (Fos)*, and *Lrp1* as the most prominent downregulated genes within these pathways (Figure 3I). Additional other cartilage regeneration‐related genes, such as bone morphogenetic protein 1 (*Bmp1), Col2α1, Col11α2, Col13α1*, and *Col27α1*, also exhibited marked downregulation in the *Wtap* KO group (Figure 3J). These results indicate that WTAP deficiency impairs stem cell chondrogenic differentiation and subsequent cartilage formation by downregulating core regulatory genes and disrupting critical signaling and metabolic pathways.

Subsequently, to further characterize the regulatory mechanisms, we used the OmicStudio platform to construct and analyze a protein‐protein interaction (PPI) network between chondrogenic genes and the three aforementioned enriched pathways. This network intuitively visualized the intricate crosstalk between the chondrogenic genes and these pathways, with a particular focus on the close interactions *Smad9, Fos, Lrp1*, and the cartilage‐related genes (Figure 3K). Such PPI network analysis clarifies the synergistic regulatory effects of these genes, which collectively govern stem cell chondrogenic differentiation to drive the complex process of cartilage regeneration.

### 2.3 Integrated Transcriptomic and Metabolomic Analysis Reveals the WTAP/LRP1/Lipid Metabolism Axis in Regulating Chondrogenic Gene Expression

**FIGURE 4:**
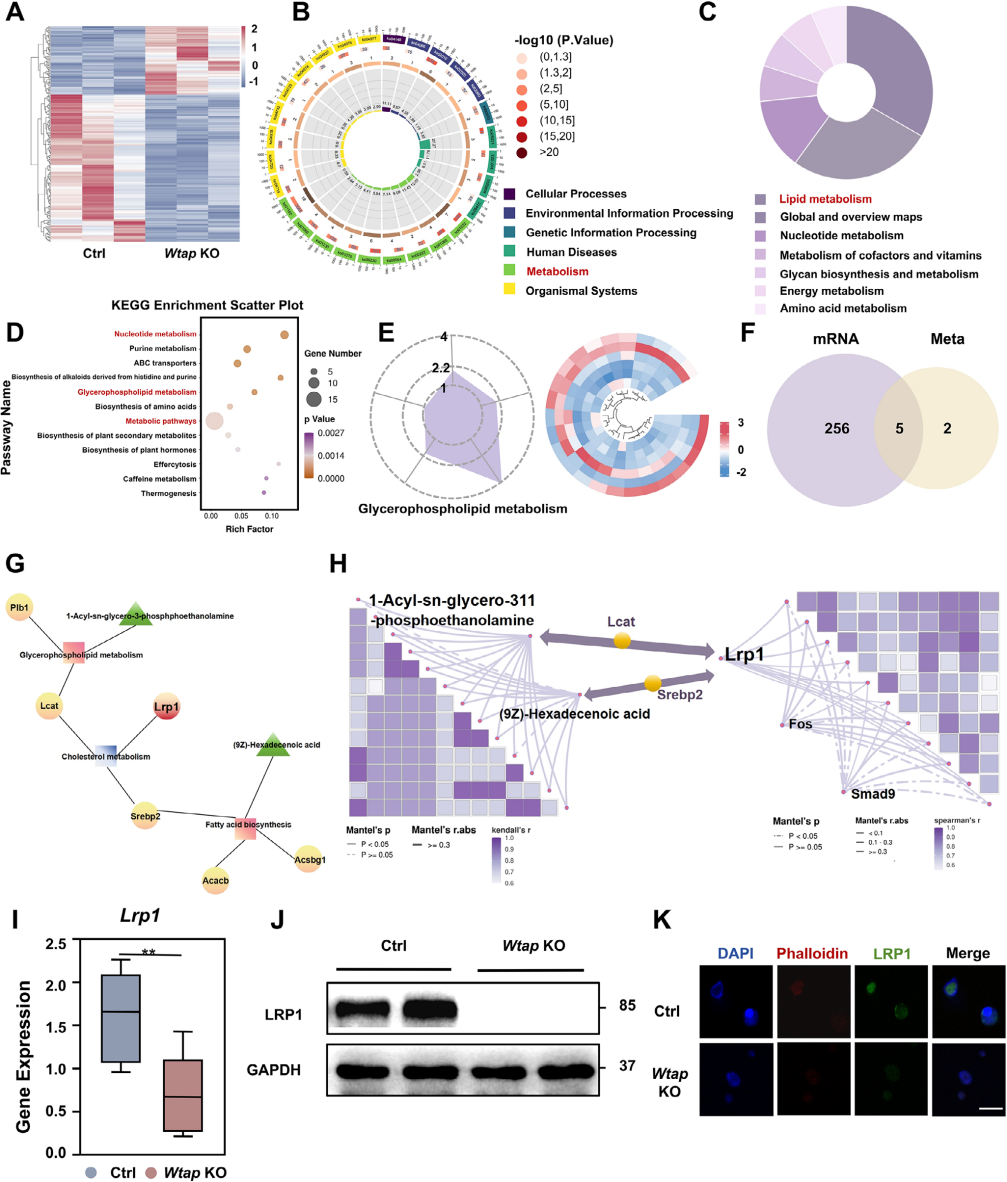
The WTAP/LRP1/lipid metabolism axis regulates chondrogenic gene expression. (A) Heatmap of DMs between the *Wtap* KO and control groups. KEGG enrichment analysis of the identified DMs (B), metabolism‐related DMs (C), and lipid metabolism‐related DMs (D) following *Wtap* knockout. (E) Radar plot of KEGG‐enriched lipid metabolism pathway (left) and the adjacent heatmap of DMs in lipid metabolism pathways (right), comparing the *Wtap* KO and control groups. (F) Venn diagram showing the overlap between DEGs and DMs from the integrated transcriptomic and metabolomic analyses. (G) Correlation network of DEGs and DMs involved in lipid metabolism pathway. (H) Correlation analysis among core genes, key metabolites, and chondrogenesis‐related genes. (I) RT‐qPCR analysis of *Lrp1* expression following *Wtap* knockout. WB results (J) and immunofluorescence (K) analysis of LRP1 protein expression in BMDMs after *Wtap* knockout (scale bar: 50 µm). Data are presented as means ± SD. ^**^ *p* < 0.01.

Building on transcriptomic findings that highlighted the critical role of metabolic processes in WTAP‐mediated cartilage regeneration, we employed liquid chromatography‐tandem mass spectrometry (LC‐MS/MS) to comprehensively characterize metabolite profiles during this process. Using the criteria of *p*‐value < 0.05, fold‐change (FC) ≥ 2 or ≤ 1/2, and variable importance projection (VIP) score ≥ 1, we identified significant differential metabolites (DMs) between the *Wtap* KO and the control groups at 2 and 4 weeks. Clustering heatmaps revealed distinct metabolic profiles between the two groups (Figure S4A; Figure 4A), reinforcing the metabolic reprogramming plays a fucriticalnctional role in WTAP‐mediated cartilage repair. KEGG enrichment analysis, presented as circle plots, clearly showed that DMs were predominantly enriched in metabolic pathways, with substantially higher representation than in organismal systems (Figure S4B; Figure 4B). In‐depth analysis of the metabolic pathways revealed that lipid metabolism ‐related pathways accounted for the largest proportion of enriched metabolic pathways at both 2 and 4 weeks (Figure S4C; Figure 4C), consistent with the transcriptomic findings. Lipid metabolism is crucial for various biological processes, including cellular energy supply, membrane composition, signal transduction, and cell growth and differentiation [28]. These findings collectively suggest that WTAP may promote the chondrogenic differentiation of stem cells by regulating key enzymes or transcription factors involved in lipid metabolism.

Subsequent detailed analysis of lipid metabolism showed enrichment in glycerophospholipid metabolism and ether lipid metabolism at 2 weeks (Figure S4D), with a shift to global metabolic pathways and glycerophospholipid metabolism at 4 weeks (Figure 4D). Glycerophospholipid metabolism and ether lipid metabolism are essential for maintaining membrane integrity and function, regulating signal transduction, and modulating inflammatory responses [29]. Notably, glycerophospholipid metabolism contained the highest abundance of DMs, among which widespread downregulation observed in the *Wtap* KO group (Figure S4E; Figure 4E). Thus, *Wtap* deficiency leads to reduced levels of DMs in lipid metabolism pathways, which may subsequently impair tissue regeneration‐related pathways and ultimately hinder the chondrogenic differentiation process of stem cells. However, the key functional targets within the lipid metabolism network that mediate cartilage regeneration remain to be identified.

To identify key hubs connecting DEGs and DMs in WTAP‐mediated cartilage regeneration, we integrated transcriptomic and metabolomic datasets. The Venn diagram showed 261 DEGs and 7 DMs, with five overlapping gene‐metabolite pairs indicating specific interactions (Figure 4F). To further elucidate the role of lipid metabolism and its potential targets, we performed correlation analysis between DEGs and DMs involved in the enriched pathways. The correlation network revealed significant associations between two DEGs—phospholipase B1 (Plb1) and lecithin‐cholesterol acyltransferase (*Lcat*)—and the DM 1‐Acyl‐sn‐glycero‐3‐phosphoethanolamine in glycerophospholipid metabolism. Similarly, in the fatty acid biosynthesis pathway, significant associations were identified between three DEGs—acetyl‐CoA carboxylase beta (*Acacb*), acyl‐CoA synthetase bubblegum family member 1 (*Acsbg1*)), and sterol regulatory element‐binding protein 2 (*Srebp2*)—and the DM (9Z)‐Hexadecenoic acid. These lipid metabolic pathways showed functional connections to cholesterol metabolism *via Lcat* and *Srebp2*, respectively (Figure 4G). Consistent with our previous transcriptomic analysis, *Lrp1* was identified as the key gene associated with cartilage regeneration in cholesterol metabolism. To confirm LRP1's regulatory role, we performed additional correlation analysis between the screened key genes/metabolites and chondrogenesis‐related genes. This analysis verified LRP1's strong associations with both key lipid metabolites and chondrogenic markers, positioning LRP1 as a central node bridging lipid metabolism and chondrogenic differentiation (Figure 4H).

To elucidate the functional relationship between WTAP and LRP1, we cultured *Wtap* WT/KO BMDMs in a GelMA hydrogel‐based 3D culture system. RT‐qPCR results demonstrated a significant reduction in *Lrp1* mRNA expression in *Wtap* KO BMDMs compared with controls (Figure 4I). Consistent with this, western blot and immunofluorescence assays confirmed a corresponding decrease in LRP1 protein level (Figure 4J,K). Thus, WTAP not only regulates *Lrp1* mRNA expression but also modulates its subsequent protein abundance. These results indicate that LRP1, a key gene in lipid metabolism, serves as a critical downstream target of WTAP‐mediated cartilage regeneration. This finding suggests that further investigation of small‐molecule drugs targeting LRP1 may provide new strategic insights for developing minimally invasive therapies for cartilage injury.

### 2.4 Human Single‐Cell Transcriptomic Analysis Supports Dysregulation of the LRP1–lipid Metabolic Axis in Osteoarthritic Macrophages

To evaluate the clinical relevance of the WTAP–LRP1 axis, we reanalyzed publicly available single‐cell RNA sequencing datasets derived from human OA and non‐OA joint tissues, including cartilage, meniscus, synovium, and infrapatellar fat pad [30]. Unsupervised clustering identified major cellular populations, among which macrophages (MAC) were markedly expanded in OA samples compared with normal tissues, indicating enhanced inflammatory infiltration (Figure S8A,D). Cell identities were validated by canonical marker gene expression (Figure S8B,C). Despite the increased abundance of macrophages in OA tissues, LRP1 expression was significantly reduced within the MAC cluster. In parallel, anti‐inflammatory and regenerative mediators, including Interleukin‑10 (IL‑10) and transforming growth factor beta 1 (TGFB1), were also downregulated in OA macrophages (Figure S8E). Gene set enrichment analysis further revealed a coordinated suppression of lipid metabolism‐related pathways—including cholesterol metabolism, ether lipid metabolism, fatty acid biosynthesis, and PPAR signaling—in the OA microenvironment (Figure S8F).

Collectively, these findings suggest that OA‐associated macrophages exhibit an LRP1‐low, metabolically suppressed phenotype, consistent with the mechanistic axis identified in our murine model.

### 2.5 FDA Screening Identifies LRP1‐Targeting Compounds Silibinin and Estradiol Benzoate With Chondrogenic Properties

**FIGURE 5:**
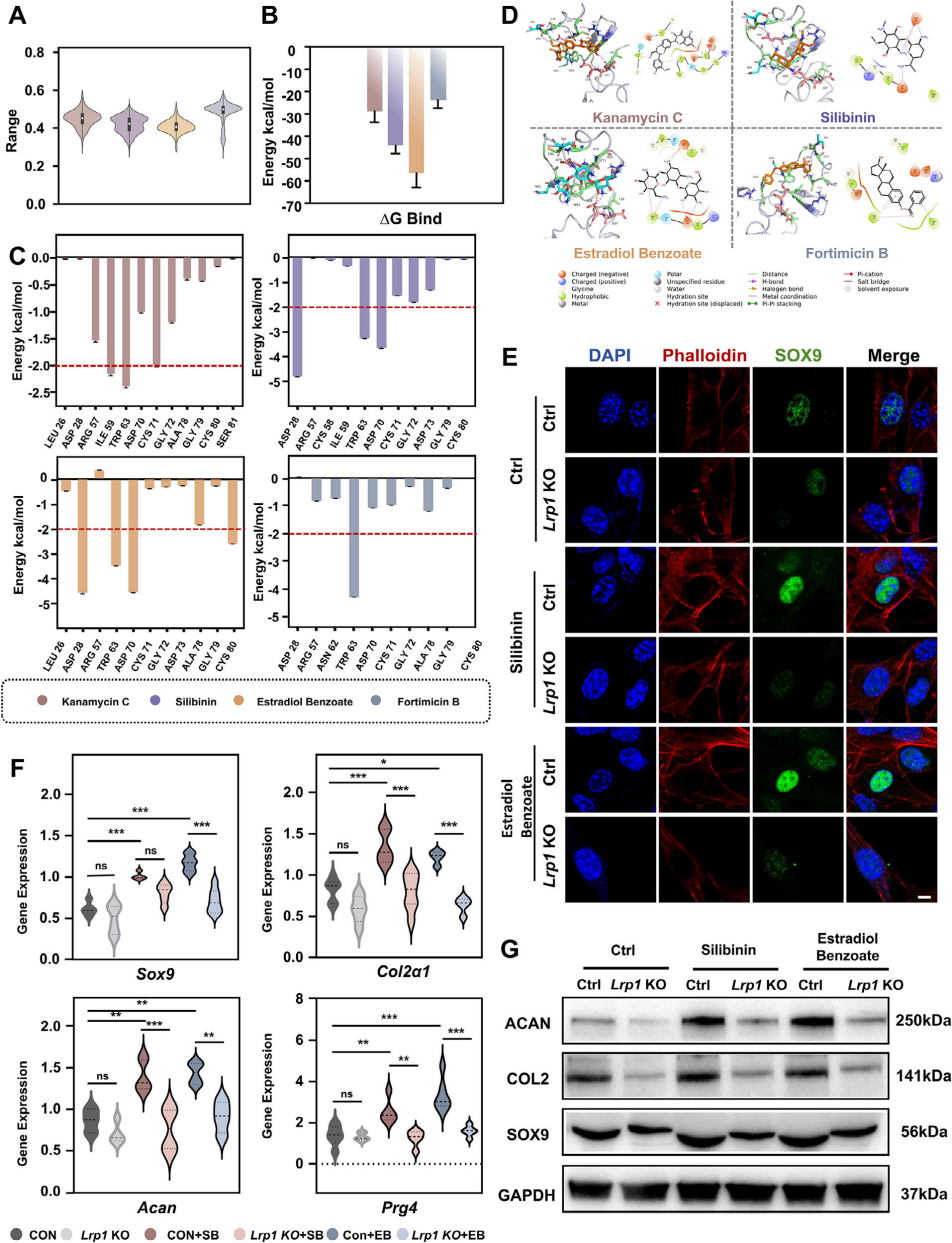
Screening identifies silibinin and estradiol benzoate as LRP1‐specific agonists enhancing chondrogenic activity. (A) RMSD analysis of LRP1 in complex with kanamycin C, silibinin, estradiol benzoate, and fortimicin B. (B) Binding free energy calculation for kanamycin C‐LRP1, silibinin‐LRP1, estradiol benzoate‐LRP1, and fortimicin B‐LRP1 complexes. (C) Per‐residue energy decomposition analysis for the four complexes. (D) Visualization of molecular interactions between four representative compounds and LRP1. (E) Immunofluorescence staining of SOX9 protein expression (green) in mBMSCs co‐cultured with WT or Lrp1 KO BMDMs following treatment with silibinin and estradiol benzoate. (scale bar: 10 µm). (F) RT‐qPCR analysis of chondrogenic genes (*Sox9, Col2α1, Acan, Prg4*) expression in mBMSCs after drug treatment. (G) WB result of chondrogenic marker protein (SOX9, COL2, ACAN) in mBMSCs from different treatment groups. Data are presented as means ± SD. ^*^ *p* < 0.05, ^**^ *p* < 0.01, ^***^ *p* < 0.001.

To develop LRP1‐targeted therapies for cartilage injury, we employed molecular dynamics (MD) simulations to screen Food and Drug Administration (FDA)‐approved drugs. Root Mean Square Deviation (RMSD) fluctuations of LRP1 complexes bound to silibinin or estradiol benzoate were generally small (Figure S5A). The median RMSD value for silibinin‐ and estradiol benzoate‐LRP1 complexes were 0.4 and 0.399 nm, respectively (Figure 5A), indicating stable binding between these two compounds and LRP1. In contrast, kanamycin C and fortimicin B showed significantly larger RMSD fluctuations when bound to LRP1. Consistently, Root Mean Square Fluctuation (RMSF) analysis showed that silibinin and estradiol benzoate reduced the atomic flexibility of LRP1, whereas kanamycin C and fortimicin B induced relatively greater fluctuations in LRP1 residues (Figure S5B). This further confirmed the high stability of the LRP1 complex with silibinin and estradiol benzoate.

Subsequently, we calculated the binding free energy (ΔG bind) to further investigate the binding affinity and stability of each compound‐LRP1 complex. As shown in Figure 5B, the ΔG bind values for silibinin and estradiol benzoate binding to LRP1 were −43.363 ± 4.26 and −55.006 ± 7.64 kcal/mol, respectively, which were significantly more favorable than those of the other two compounds. We further analyzed the energy contributions of amino acid residues within a 6Å radius of the protein surface to identify key residues involved in molecular recognition and binding. The number of amino acids contributing over −2 kcal/mol to the binding of silibinin and estradiol benzoate to LRP1 was substantially higher than for the other two compounds (Figure 5C), indicating stronger residue‐level interactions and thus greater binding stability. To elucidate the interaction mechanisms, we analyzed the 3D binding modes and specific interactions of the candidate compounds with LRP1. Silibinin and estradiol benzoate formed salt bridges between their protonated amino groups and the acidic, negatively charged amino acids aspartic acid 70 (Asp 70) and Asp28 in the LRP1 protein (Figure 5D). In summary, silibinin and estradiol benzoate exhibit highly specific and stable binding to LRP1 protein, providing a crucial molecular basis for their potential as targeted small‐molecule drugs for cartilage injuries.

We next evaluated the biosafety of silibinin and estradiol benzoate using live/dead cell staining. After 1, 2, and 3 days of drug stimulation, abundant live cells (green fluorescence) were observed in all groups (Figure S6A), indicating that neither compound affected BMDM viability compared with the control. Similarly, the Cell Counting Kit‐8 (CCK‐8) assay showed no significant reduction in optical density (OD) values in the compound‐treated groups relative to the control (Figure S6B), further confirming the favorable biosafety profiles of both silibinin and estradiol benzoate. To verify whether the chondrogenic effects of these compounds depend on LRP1, we successfully generated the *lrp1* KO BMDM via lentiviral transduction (Figure S7A,B). And then, we added the two small molecules to *Lrp1* WT/KO BMDMs, which were co‐cultured with mBMSCs to simulate a cartilage regeneration microenvironment. RT‐qPCR results showed that, following silibinin or estradiol benzoate treatment, the mRNA expression levels of chondrogenesis‐related genes (*Sox9, Col2α1, Acan*, and *Prg4*) were significantly lower in the *Lrp1* KO group compared than in the control (Figure 5F). Immunofluorescence staining revealed weaker green fluorescence intensity of the chondrogenic marker SOX9 in mBMSCs co‐cultured with *Lrp1* KO BMDMs (Figure 5E). Similarly, western blot analysis demonstrated that the protein expression levels of the chondrogenic markers SOX9, COL2, and ACAN were markedly reduced in *Lrp1* KO group compared with the control after compound stimulation (Figure 5G). These findings demonstrate that the promotive effects of silibinin and estradiol benzoate on mBMSC chondrogenic differentiation are significantly abrogated in the absence of LRP1. This conclusively confirms that silibinin and estradiol benzoate exert their chondrogenic effects on mBMSCs by directly binding to LRP1.

### 2.6 Silibinin and Estradiol Benzoate Promote Cartilage Regeneration In Vivo

**FIGURE 6:**
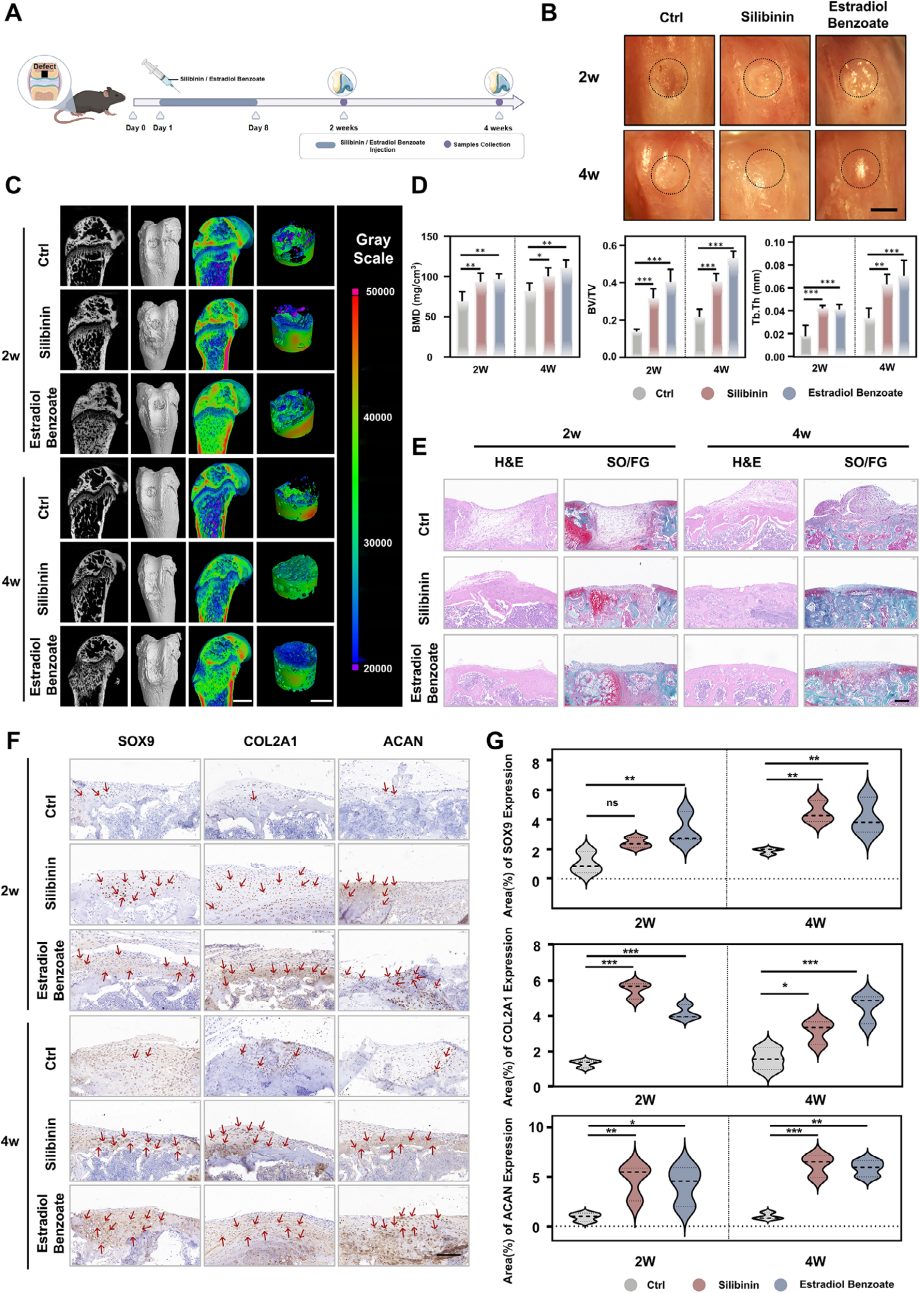
Silibinin and estradiol benzoate promote cartilage regeneration in vivo. (A) Schematic diagram of the cartilage defects model and experimental treatment method. (B) Representative photographs of knee joint after different treatments at 2 and 4 weeks. (C) Representative micro‐CT images and 3D reconstructions of subchondral bone. Scale bar: 2 mm (left), 0.5 mm (right). (D) Quantitative micro‐CT analysis of BMD, BV/TV, and Tb. The in drug‐treated versus control groups. (E) H&E staining and safranin‐O fast green staining of the knee joint tissue section (scale bar: 100 µm). (F) Immunohistochemical staining of SOX9, COL2A1, and ACAN in knee joint tissue section (*n* = 5; scale bar: 100 µm). (G) Quantification of the percentage of positively stained areas for SOX9, COL2A1, and ACAN is shown in (F). Data are presented as means ± SD. ^*^ *p* < 0.05, ^**^ *p* < 0.01, ^***^ *p* < 0.001.

Building on our in vitro findings that silibinin and estradiol benzoate promote chondrogenesis via LRP1 binding, we established a mouse model of full‐thickness osteochondral defects in the knee joint to investigate their effect of cartilage regeneration in vivo. Post‐surgery, mice received daily intra‐articular injections of 1% dimethyl sulfoxide (DMSO) (the control), silibinin, or estradiol benzoate for 7 days, and repair outcomes were evaluated at 2 and 4 weeks. (Figure 6A).

Gross morphological assessment revealed markedly superior cartilage healing in the silibinin and estradiol benzoate groups compared with the control, indicating a short‐term promotive effect on cartilage repair. By 4 weeks, cartilage defects in the drug‐treated groups exhibited near‐complete repair with smooth articular surfaces, confirming sustained regenerative activity (Figure 6B). Consistently, micro‐CT scanning and 3D reconstruction demonstrated increased new bone formation within the cartilage defect area in the drug‐treated groups, verify their efficacy in promoting subchondral bone regeneration (Figure 6C). Quantitative micro‐CT analysis showed that the drug‐treated groups had significantly higher bone mineral density (BMD), bone volume/total volume (BV/TV) ratio, and trabecular thickness (Tb. Th) compared with the control (Figure 6D). These results reflect improved microstructure of the newly formed bone, suggesting that silibinin and estradiol benzoate promote the formation of healthier, more robust osseous structure.

Histological analyses further supported these findings. H&E staining showed that the cartilage defect area was significantly reduced in the drug‐treated groups, with new bone tissue covering the defect surface. In contrast, the control group defects were primarily filled with fibrous connective tissue and minimal new bone. Safranin O‐Fast Green staining results revealed more newly formed cartilage, abundant chondrocytes, and more intact knee joint morphology in the drug‐treated groups (Figure 6E), confirming their superiority in promoting neocartilage formation. Immunohistochemical (IHC) staining showed markedly more dark brown granules—representing positive expression of the chondrogenic markers SOX9, COL2A1, and ACAN—in the cytoplasm and extracellular matrix of healing sites in the drug‐treated groups related to the control. (Figure 6F). This indicates enhanced chondrocyte activity and increased cartilage matrix synthesis. Collectively, these in vivo findings provide a solid scientific basis for the potential application of silibinin and estradiol benzoate in targeted cartilage injury therapy. Our study demonstrates that WTAP regulates cartilage regeneration through a cascade centered on the WTAP‐LRP1‐lipid metabolism axis, and silibinin and estradiol benzoate can target LRP1 to promote cartilage repair. This provides a novel epigenetic strategy for developing LRP1‐targeted minimally invasive therapies for cartilage injury.

## 3 Discussion

Epigenetic RNA modifications are increasingly recognized as key regulators of tissue repair [31], however, the specific epitranscriptomic mechanisms governing cartilage regeneration remain unclear. In this study, we identify WTAP‐mediated m^6^A modification as an essential driver of chondrogenic differentiation and cartilage repair. Using in vitro mBMSC differentiation assays and an in vivo knee defect model, we demonstrate that loss of WTAP markedly impairs cartilage formation capacity. Integrated RNA‐seq and metabolomic analyses further reveal that WTAP deficiency disrupts lipid metabolic programs, particularly glycerophospholipid metabolism, and suppresses cartilage‐associated gene expression. Based on these mechanistic insights, we performed molecular docking‐based virtual screening to identify LRP1 agonists, thereby confirming the functional relevance and therapeutic potential of the WTAP–LRP1 axis. Collectively, these findings establish WTAP as a critical epigenetic mediator linking m^6^A‐dependent RNA regulation with metabolic pathways central to cartilage regeneration.

WTAP has long been recognized as an essential structural component of the METTL3/METTL14 m^6^A methyltransferase complex and is required for proper catalytic localization and substrate recognition [19]. Although previous studies have primarily examined WTAP in cancer biology [32, 33] and embryonic development [34, 35], its functional relevance in tissue regeneration remains poorly defined. Building upon our prior work implicating WTAP in immuno‐skeletal metabolic regulation [21], the present study expands the biological scope of WTAP by demonstrating that its m^6^A‐dependent RNA regulatory activity directly shapes the metabolic landscape required for cartilage formation. Notably, our findings differ from earlier studies emphasizing METTL3‐mediated m^6^A modification in chondrogenesis [20], in which METTL3 was shown to reduce the expression of autophagy‐related 7 by destabilizing its RNA [36]. In contrast, we identify WTAP as an upstream determinant that stabilizes transcripts involved in lipid and cholesterol metabolism. This establishes a previously unrecognized epigenetic–metabolic axis through which WTAP orchestrates chondrogenic differentiation, linking m^6^A signaling to lipid‐driven anabolic pathways in cartilage regeneration.

Because complex biological processes are orchestrated through coordinated transcriptional and metabolic regulation [37], single‐omics approaches often lack sufficient resolution to identify key functional nodes [38, 39, 40]. Accordingly, integrated transcriptomic and metabolomic analyses revealed LRP1 as a pivotal lipid‐metabolic regulator in WTAP‐driven chondrogenesis. LRP1 is increasingly recognized as a multifunctional receptor integrating lipid metabolism [41], cellular signaling [42], and tissue homeostasis [43]. In cartilage biology, previous studies have shown that LRP1 deficiency impairs chondrogenic differentiation and disrupts anabolic signaling pathways, underscoring its importance in maintaining cartilage integrity [44]. Specifically, LRP1 plays a crucial dual role in joint protection: it promotes chondrocyte maturation via TGF‐β1/WNT signaling [45] and prevents extracellular matrix degradation through the endocytic clearance of key catabolic enzymes, such as a disintegrin and metalloproteinase with thrombospondin motifs 5 (ADAMTS‑5) and matrix metalloproteinase‑13 (MMP‑13) [46]. While these findings highlight LRP1 as a vital therapeutic target for driving cartilage regeneration in OA, the epigenetic regulation of LRP1 during this process remains largely unexplored. Our findings position LRP1 as a critical downstream effector of WTAP‐mediated m^6^A modification, thereby linking RNA epigenetic regulation to lipid‐metabolic control of chondrogenesis. By stabilizing *Lrp1* transcripts, WTAP enables the activation of lipid‐metabolic programs that support matrix synthesis and chondrocyte differentiation. This regulatory relationship places LRP1 within an epigenetic–metabolic framework and identifies that precise control of LRP1 expression is a key mechanism through which m^6^A signaling governs cartilage regeneration. Importantly, our reanalysis of human single‐cell datasets provides a clinical context for the WTAP–LRP1 regulatory axis. Although macrophages were expanded in OA tissues, these cells exhibited reduced LRP1 expression and suppression of lipid metabolic programs. Given the established role of lipid metabolism in macrophage polarization and tissue repair, these findings suggest that OA‐associated macrophages may exist in a metabolically constrained and functionally impaired state. The consistency between the human transcriptomic data and our mechanistic findings in murine models supports the translational relevance of targeting the WTAP–LRP1–metabolic pathway in cartilage regeneration.

The central role of LRP1 within the WTAP‐mediated epigenetic–metabolic axis suggests that this receptor represents a tractable target for pharmacological intervention. Through structure‐based virtual screening of FDA‐approved compounds, we identified silibinin and estradiol benzoate as effective LRP1 agonists. Although both agents have been clinically used for metabolic or endocrine indications [47, 48, 49], their roles in cartilage regeneration have not been established. Our findings demonstrate that the pro‐chondrogenic and reparative effects of pharmacological LRP1 activation are significantly attenuated upon *Lrp1* depletion. Notably, despite their distinct chemical structures, silibinin and estradiol benzoate exhibited no statistical difference in either LRP1‐binding stability or their efficacy in promoting chondrogenesis. This dual‐candidate outcome provides versatile options for future clinical translation. Furthermore, combination drug therapy is increasingly recognized for their potential to reduce individual drug dosages, mitigate dose‐dependent toxicity, and enhance overall therapeutic depth [50]. Therefore, future studies should explore the synergistic potential of a low‐dose combination strategy employing both agents to maximize chondrogenic efficacy while minimizing potential systemic side effects. Beyond the selection of therapeutic agents, the delivery strategy is equally critical. The early phase of cartilage repair represents a vital temporal window that dictates the ultimate quality of tissue regeneration [51]. Given the rapid intra‐articular clearance of small molecules, our study employed a regimen of daily intra‐articular injections for seven consecutive days post‐surgery to maintain an adequate and stable local drug concentration during this pivotal period. However, from a translational perspective, frequent intra‐articular injections inevitably increase the risk of infection and severely compromise patient compliance. To overcome this hurdle, future investigations should focus on integrating these small‐molecule agonists with advanced biomaterial platforms. Utilizing in situ hydrogels [52], sustained‐release microspheres [53], or bioresponsive nanocarriers [54] could significantly optimize drug release kinetics, thereby achieving long‐term, spatiotemporally controlled targeted delivery within the joint cavity.

Overall, our research establishes the WTAP–LRP1 axis as an epigenetic–metabolic cascade that promotes cartilage regeneration by stabilizing LRP1 and activating downstream lipid‐metabolic programs. Molecular docking‐based screening of LRP1‐targeting compounds, including silibinin and estradiol benzoate, further provides a feasible therapeutic strategy for targeting epigenetic–metabolic dysregulation in OA.

## 4 Conclusion

In summary, this study elucidated the epigenetic‐metabolic regulatory mechanism underlying WTAP‐mediated m^6^A modification in OA‐related cartilage regeneration. Our findings demonstrated that WTAP enhances m^6^A methylation, thereby orchestrating chondrogenesis through *Lrp1* transcripts and promoting a pro‐regenerative reprogramming of lipid metabolism. Leveraging this target, structure‐based screening yielded silibinin and estradiol benzoate as specific LRP1 agonists. These compounds potently activate the WTAP‐LRP1 pathway, facilitating robust cartilage repair in vivo. This study highlights the critical intersection between epigenetic regulation and metabolism in cartilage regeneration. This fundamental insight unlocks a novel rationale for designing targeted drug delivery systems for OA, potentially achieving unprecedented levels of cartilage regenerative efficacy.

## 5 Experimental Section

### 5.1 Mouse Model of Knee Joint Cartilage Defect

C57BL/6 mice were obtained from Peking University Health Science Center's Laboratory Animal Science Department. All experiments followed ethical guidelines and were approved by the Institutional Animal Care and Use Committee (Approval No.: PUIRB‐LA2024015). A knee joint cartilage defect model was established in 6‐ to 8‐week‐old mice (weighing approximately 20 g). Mice were anesthetized via intraperitoneal injection of 0.15 mL sodium pentobarbital (10 mg/mL). The surgical site was shaved, disinfected with iodophor, and locally anesthetized with 0.5% lidocaine hydrochloride. An incision was made to expose the patellar groove, and a blunted syringe needle (0.8 mm diameter, 1 mm depth) was used to create a cartilage defect in the femur. After surgery, all experimental mice were housed under standard conditions with unrestricted access to food and water, and allowed free movement with adequate rest.

### 5.2 Cell Culture

Primary mouse BMDMs were purchased from iCell Bioscience Inc. (Shanghai, China). BMDMs were cultured in high‐glucose DMEM medium (Procell, China) supplemented with 10% fetal bovine serum (FBS) and 1% penicillin‐streptomycin. Cells were used for experiments upon reaching 80% confluence and maintained in a constant temperature environment of 37°C with 5% CO_2_. Primary mBMSCs were purchased from Procell Life Science & Technology Co., Ltd. (Wuhan, China). The mBMSCs were cultured in αMEM medium (Procell, China) containing 10% FBS and 1% penicillin‐streptomycin. Cells were utilized for experiments when they reached 80% confluence and maintained under the same standard conditions (37°C, 5% CO_2_).

### 5.3 Lentiviral Transduction

BMDM cell suspension were prepared in complete medium at a density of 3–5 × 10^4^ cells/mL and seeded into 6‐well plates. After culturing at 37°C with 5% CO_2_ for 16–24 h, cells reached 20%–30% confluence. Following PBS washing, each well of the experimental groups was treated with 200 µL of *Wtap/Lrp1* lentivirus (Genechem, China) and 40 µL of HiTransG P enhancer, and the mixture was adjusted to a final volume of 1 mL with high‐glucose DMEM. The control group received fresh complete medium instead. After 12–16 h of incubation at 37°C, the medium was replaced with regular culture medium. Cells were then cultured for 3 days, during which the medium was changed, or subculturing was performed as needed. At 72 h post‐infection, when cells reached with 80% confluence, they were maintained in culture medium containing 5 µg/mL puromycin (Solarbio, China), with the medium replaced every 3–4 days. Wild‐type non‐transduced cells served as negative controls and were completely eliminated by puromycin selection, whereas virus‐transduced cells exhibited no further cell death. Fluorescence microscopy revealed nearly 100% green fluorescence in the transduced group, confirming successful lentiviral transduction.

### 5.4 Preparation of BMDM‐Laden GelMA Hydrogel and In Vivo Injection

High‐glucose DMEM medium (20 µL) was mixed with 0.05 mg of the photo‐initiator LAP (EFL, China) in a brown glass bottle, followed by heated at 40°C–50°C for 15 min with constant stirring. One gram of GelMA60 was then added to this solution in a centrifuge tube and heated at 60°C–70°C for 20 min in the dark while stirring. The resulting GelMA solution was sterilized using a 0.22 µm syringe filter. Cultured *Wtap* KO/WT BMDMs were resuspended in the GelMA solution (prewarmed to 37°C) to achieve a cell density of 1 × 10^6^ cells/mL. This cell‐laden GelMA suspension was loaded into a syringe and photo‐crosslinked for 20 s using a UV light source. Thirty‐two mice were randomly divided into the control group and the *Wtap* KO group, and the hydrogel constructs were injected into the knee joint cartilage defects of each mouse. Samples were collected 2 and 4 weeks. For sample collection, the 32 mice were euthanized, and tissue samples from their knee joint cartilage defects were harvested. Among these samples, those from 22 mice were flash‐frozen for transcriptomic and metabolomic sequencing, while the remaining 10 samples were processed for histological analysis.

### 5.5 Transwell Co‐Culture and Chondrogenic Differentiation

GelMA hydrogel (GelMA60, EFL, China) was used as a carrier, and 12‐well Transwell plates were employed to culture *Wtap* WT/KO BMDMs. BMDMs were resuspended in GelMA solution at a density of 3 × 10^6^ cells/mL, and 200 µL of this cell suspension was photo‐crosslinked in the Transwell insert. Subsequently, 500 µL of culture medium was added to each well. mBMSCs were seeded in the lower chamber at 3 × 10^4^ cells/mL. Once mBMSCs reached 60% confluence, chondrogenic induction medium (Cyagen, China) was added to both chambers, which was refreshed every 2 days. When mBMSCs reached 90% confluence, they were subcultured. The co‐culture lasted 14 days before sample collection.

### 5.6 Transcriptome Sequencing and Analysis

Total RNA was extracted from animal tissues using TRIzol, and its concentration and purity were measured with a NanoDrop 8000 (Thermo Fisher Scientific, USA). RNA integrity was assessed using an Agilent Bioanalyzer 2100. mRNA was isolated with Oligo(dT) beads (Thermo Fisher Scientific, USA), fragmented into short segments, and reverse‐transcribed into first‐strand cDNA. Double‐stranded cDNA was subsequently synthesized, and strand‐specific libraries were constructed via dUTP labeling. After end repair, adenine‐tailing, and adapter ligation, cDNA fragments were selected to target an insert size of 300 ± 50 bp. Libraries were then processed for sequencing on an Illumina Novaseq 6000 (LC‐Bio, Hangzhou, China) using a paired‐end 150 (PE150) strategy, with subsequent data analysis conducted on the OmicStudio platform.

### 5.7 Metabolome Sequencing and Analysis

Samples were thawed on ice, and metabolites were extracted using 50% methanol buffer. Each sample (20 µL) was mixed with 120 µL of chilled 50% methanol, vortexed for 1 min, and incubated at room temperature for 10 min. The mixture was stored at –20°C overnight. After centrifugation at 4,000 g for 20 min at 4°C, the supernatant was transferred to a 96‐well plate and stored at –80°C until it was processed for LC‐MS analysis. A pooled quality control (QC) sample was prepared by combining 10 µL of each extract. LC‐MS analysis was conducted using a Thermo Scientific UltiMate 3000 HPLC system coupled with an ACQUITY UPLC BEH C18 column (100 mm ^*^ 2.1 mm, 1.8 µm; Waters, UK) maintained at 35°C. The mobile phase included solvent A (water with 0.1% formic acid) and solvent B (acetonitrile with 0.1% formic acid), with a flow rate of 0.4 mL/min. The gradient elution program was as follows: 0–0.5 min, 5% B; 0.5–7 min, 5% to 100% B; 7–8 min, 100% B; 8–8.1 min, 100% to 5% B; 8.1–10 min, 5% B. The injection volume was 4 µL. Metabolites eluted from the column were analyzed using a Q‐Exactive mass spectrometer in both positive and negative ion modes. Full‐scan MS spectra (m/z 70–1050) were recorded at a resolution of 70,000, with an automatic gain control (AGC) target of 3e^6^ and an injection time of 100 ms. Data‐dependent acquisition (DDA) was performed using a top‐3 method, with MS/MS spectra acquired at a resolution of 17 500, an AGC target of 1e^5^, and an injection time of 80 ms. A pooled QC sample was run every 10 samples to ensure instrument stability.

### 5.8 Integrated Transcriptomic and Metabolomic Analysis

Low‐quality sequences were removed from both transcriptomic and metabolomic datasets. followed by data normalization for initial quality control. Differentially expressed genes and differential metabolites were identified, and KEGG pathway analysis was used to integrate data, highlighting significant changes in biological processes. Network analysis was performed to construct an interaction network between the datasets. Correlation analysis on the OmicStudio platform, using Pearson coefficients, clarified relationships between differential genes and metabolites. These integrated analyses facilitated targeted investigation of key regulatory genes involved in the core biological pathways.

### 5.9 Tissue Embedding and Sectioning

Bone samples were fixed in 4% paraformaldehyde (Servicebio, China) for 24 h, rinsed with PBS (Solarbio, China), and decalcified in 10% EDTA (Servicebio, China) for 7 days with daily solution changes. Following decalcification, the samples were dehydrated through a graded ethanol series (Sinopharm, China), cleared in xylene (Sinopharm, China), and embedded in paraffin. Subsequently, they were sectioned into 4 µm slices using a microtome. The sections were mounted on slides, dried, deparaffinized in xylene, and finally rehydrated through a graded alcohol series.

### 5.10 H&E Staining

The sections were stained with hematoxylin (Baso, China) for 3–5 min, rinsed with distilled water, differentiated, and blued with a bluing reagent (Servicebio, China). After rinsing, they were dehydrated through graded alcohol, counterstained with eosin (Baso, China) for 5 min, and cleared in xylene. Finally, the sections were mounted with neutral balsam (Sinopharm, China) and examined under a microscope (Nikon, Japan).

### 5.11 Safranin O‐Fast Green Staining

Following deparaffinization and dehydration, sections were stained with Fast Green (Servicebio, China) for 1–5 min and rinsed with distilled water until the cartilage became colorless. They were then briefly treated with 1% hydrochloric acid, rinsed, stained with Safranin O (Servicebio, China) for 5–10 s. Rapid dehydration was performed through four changes of ethanol, each for 3–5 s, until the cartilage turned red and the background cleared. Finally, sections were cleared in xylene twice (5 min each) and mounted with neutral balsam for microscopic examination and imaging.

### 5.12 Immunohistochemical Staining

Following antigen retrieval in EDTA buffer (Servicebio, China), tissue sections were blocked with 3% BSA (Servicebio, China) for 30 min and then incubated with primary antibodies overnight at 4°C. After washing with PBS, the sections were incubated with a secondary antibody for 1 h. DAB substrate was applied until brownish‐yellow coloration developed, followed by a rinse with deionized water. The sections were counterstained with hematoxylin for 3 min, dehydrated, cleared with xylene, mounted, and then examined under a microscope. The primary antibodies used included: SOX9 (1:500, ab185966, Abcam), ACAN (1:2000, ab313636, Abcam), and COL2A1 (1:400, ab34712, Abcam).

### 5.13 Reverse Transcription Quantitative Polymerase Chain Reaction (RT‐qPCR)

Total RNA was extracted from BMSCs and BMDMs using TRIzol reagent (Sigma, USA). cDNA was then synthesized from the RNA. Quantitative PCR was performed on a QuantStudio 3 system (Thermo Fisher Scientific, USA) using a SYBR Green RT‐PCR kit (AG, China). All samples were analyzed in triplicate. The primer sequences used are listed in Table S1.

### 5.14 Western Blotting

BMSCs and BMDMs were lysed using RIPA buffer containing protease inhibitors (Beyotime, China). Because the massive glycosylation of intact aggrecan (ACAN) hinders its electrophoretic migration, specific protein samples underwent enzymatic deglycosylation prior to SDS‐PAGE. Briefly, 30 µg of protein extracts was incubated with Chondroitinase ABC (0.1 U/mL, Sigma–Aldrich) in deglycosylation buffer (50 mm Tris‐HCl, 50 mm sodium acetate, pH 8.0) at 37°C for 2 h to cleave the chondroitin sulfate chains. Post‐digestion, the lysates were mixed with loading buffer and boiled. Proteins were separated by SDS‐PAGE (12% gels for SOX9 and COL2A1; 8% gels were utilized to resolve the high‐molecular‐weight ACAN) and transferred onto PVDF membranes (Sigma, USA). After blocking with 5% non‐fat milk in TBST (Solarbio, China), the membranes were incubated with primary antibodies overnight at 4°C, followed by incubation with HRP‐conjugated secondary antibodies (1:1000, Beyotime, China) for 1 h at 37°C. Following the wash, signals were detected using enhanced chemiluminescence and visualized with a Bio‐Rad imaging system. The primary antibodies used were as follows: SOX9 (1:10000, ab185966, Abcam), ACAN (1:10000, ab313636, Abcam), and COL2A1 (1:10000, ab34712, Abcam). Following the deglycosylation procedure, the ACAN core protein was clearly detected at a molecular weight of approximately 250 kDa.

### 5.15 Immunofluorescence

After 14 days of chondrogenic induction, the Transwell inserts were removed. mBMSCs from the lower chamber were then seeded into confocal dishes and cultured for one day at 37°C with 5% CO_2_. For immunofluorescence staining, the cells were washed with PBS, fixed with 4% paraformaldehyde for 30 min, permeabilized with 0.1% Triton X‐100 (Solarbio, China), and blocked with 3% BSA. They were incubated overnight at 4°C with a primary antibody against SOX9 (1:1000, ab185966, Abcam). After washing, the cells were incubated with an Alexa Fluor 488 secondary antibody (1:200, ab150077, Abcam) for 1 h at room temperature. Following PBS washes, the actin cytoskeleton was stained with rhodamine‐phalloidin (1:200, CA1610, Solarbio) for 30 min at room temperature. Nuclei were then counterstained with DAPI (1:1000, C0060, Solarbio) for 10 min, followed by three additional PBS washes. Finally, samples were preserved in PBS and examined using a Leica confocal microscope (Leica, Germany), with images captured and analyzed via Leica X software.

### 5.16 Molecular Dynamics Simulation

Molecular dynamics simulations of the target protein LRP1 were performed using GROMACS 5.1.5. The Amber14SB force field and TIP3P solvent model were employed, with AM1‐BCC charges and GAFF parameters assigned to the small molecules. The system was neutralized with chloride ions and energy‐minimized using steepest descent followed by conjugate gradient methods. After equilibration at 300 K and 101.325 kPa for 100 ps, a production run of 50 ns was conducted. Conformational dynamics were analyzed by calculating the root‐mean‐square deviation (RMSD) and root‐mean‐square fluctuation (RMSF) with GROMACS tools, and principal component analysis (PCA) was performed on the alpha‐carbon atoms using the gmx covar module.

### 5.17 Binding Free Energy Calculation

The binding free energy (ΔG bind) between the target protein LRP1 and candidate drugs was calculated using the Molecular Mechanics/Generalized Born Surface Area (MM/GBSA) method. The binding energy was subsequently calculated from the molecular dynamics trajectories using the gmx_MMPBSA program.

### 5.18 Binding Mode Analysis

The crystal structure of the LRP1 protein‐ligand complex was retrieved from the Protein Data Bank and prepared using PyMOL by removing water molecules, co‐solvents, and non‐essential chains. The ligand was extracted and saved separately. Multiple 3D conformations of the ligand were generated with RDKit. The protein structure was optimized through the removal of extraneous chains, repairing residues, and the addition of hydrogen atoms. The active site was identified based on known binding regions or functional domains. Docking studies were performed using AutoDock Vina, with binding affinity assessed by calculating the interaction energy across sampled ligand conformations. Finally, a re‐docking analysis was carried out to verify the docking experiment's accuracy by comparing the optimal ligand binding pose with the original crystal structure to evaluate whether the native position and orientation could be accurately reproduced.

### 5.19 CCK‐8 Assay

Cell proliferation was assessed using a CCK‐8 kit (Solarbio, China) on BMDMs treated with silibinin and estradiol benzoate. The experimental groups included: control (1% DMSO), silibinin (5 mg/mL, Macklin, China), and estradiol benzoate (4 µg/mL, Macklin, China). BMDMs were prepared in high‐glucose DMEM at 3 × 10^4^ cells/mL and seeded into a 96‐well plate (500 µL per well, three replicates per group). After incubation at 37°C with 5% CO_2_ for 1, 2, and 3 days, the cells were washed with PBS, and a CCK‐8 solution (1:10 CCK‐8 reagent to medium) was added (300 µL per well). Following 2 h incubation, the reaction solution was transferred to a new plate (100 µL per well, three replicates), and OD at 450 nm was measured with a microplate reader (Tecan, Germany). The obtained OD values were statistically analyzed.

### 5.20 Live/Dead Cell Staining

A live/dead cell staining assay was performed to evaluate the in vitro cytotoxicity of silibinin and estradiol benzoate. BMDMs were prepared as a cell suspension and divided into three groups: control (1% DMSO), silibinin (5 mg/mL), and estradiol benzoate (4 µg/mL). Cells were adjusted to a density of 3 × 10^4^ cells/mL in high‐glucose DMEM containing the corresponding drug concentrations and seeded into a 24‐well plate (three replicates per group). After incubation at 37°C with 5% CO_2_ for 1, 2, and 3 days, the cells were washed, and  500 µL of PBS with 4 µm calcein‐AM and 16 µm propidium iodide (PI, Solarbio, China) was added. Following incubation for 30 min at room temperature incubation, the cells were washed again, and live (green) and dead (red) cells were visualized and photographed using a fluorescence microscope (Olympus, Japan).

### 5.21 Intra‐Articular Drug Injection in Mice

Thirty mice were randomly assigned to three groups: 1) control group (1% DMSO); 2) silibinin group (100 mg/mL); 3) estradiol benzoate group (6 mg/mL). A cartilage defect model was established in the femoral knee joint as described previously. Animals were identified by ear notching and received oral penicillin post‐operatively to prevent infection. Starting on postoperative day 2, 10 µL of the corresponding solution (either 1% DMSO, silibinin at 100 mg/mL, or estradiol benzoate at 6 mg/mL) was injected into the joint cavity of the surgical site every day for a week.

### 5.22 Micro‐CT Analysis

Mouse knee joint samples were fixed in 4% paraformaldehyde for 24 h, rinsed with PBS, and stored in PBS at 4°C. The defect regions were scanned using a micro‐computed tomography (micro‐CT, SIEMENS, Germany). 3D reconstruction was performed with Cobra software, and resulting images were analyzed using the Inveon Research Workplace V 2.2.0 workstation to quantify the follow parameters in the defect area, including bone mineral density (BMD), bone volume/tissue volume (BV/TV), and trabecular thickness (Tb. Th).

### 5.23 Single‐Cell RNA Sequencing (scRNA‐seq) Data Acquisition and Processing

Publicly available human single‐cell RNA‐seq datasets were obtained from the Gene Expression Omnibus (GEO) database. The datasets comprised normal and osteoarthritic (OA) joint tissues, including cartilage (GSE220243, GSE169454, GSE255460), meniscus (GSE220243), and synovium with infrapatellar fat pad (GSE216651). Raw count matrices were processed using the Seurat R package (version 4.3.0). Quality control was performed to filter out low‐quality cells based on the number of expressed genes, unique molecular identifiers (UMIs), and the percentage of mitochondrial genes. Following quality control, data were normalized and scaled, and highly variable genes were identified for downstream analysis. To eliminate batch effects across different datasets and biological replicates, data integration was conducted using the Seurat integration anchors algorithm. Principal component analysis (PCA) was performed on the integrated dataset, followed by Uniform Manifold Approximation and Projection (UMAP) for non‐linear dimensionality reduction and visualization.

### 5.24 Statistical Analysis

Statistical analyses in this study were performed using GraphPad Prism software (version 7.0). Quantitative data are presented as the mean ± standard deviation (Mean ± SD). Comparisons between two groups were conducted using the unpaired Student's *t*‐test. Differences among multiple groups were assessed by one‐way analysis of variance (one‐way ANOVA). A *p*‐value of less than 0.05 was considered statistically significant, while a *p*‐value of less than 0.01 was considered highly statistically significant (^*^p < 0.05, ^**^p < 0.01, and ^***^p < 0.001).

## Author Contributions

C. H., C. D., Z. G., and C. C. contributed equally to this work. C. H., C.D., Z. G., and C. C. conducted the experiments and performed data analysis. H. Z contributed to material synthesis and characterization. H. Z. and C. C assisted with in vivo experiments and histological evaluations. C. H., Z. G. and C. D. contributed to data interpretation and figure preparation. Y. Y. and Y. W. designed the research and supervised the project. All authors contributed to writing the paper.

## Conflicts of Interest

The authors declare no conflicts of interest.

## Supporting information




**Supporting File 1**: advs75479‐sup‐0001‐SuppMat.docx.

**Supporting File 2**: advs75479‐sup‐0002‐FigureS1‐S8.zip.

## Data Availability

The data that support the findings of this study are available from the corresponding author upon reasonable request.
