# Supplementary material for: WTAP‐Mediated m6A Modification Targets the LRP1‐Lipid Metabolism Axis to Regulate Joint Cartilage Regeneration
Source: Adv Sci (Weinh). 2026 May 6;13(41):e75479. doi: 10.1002/advs.75479 (PMC13335596; doi:10.1002/advs.75479)
Supplement: Supplementary file 1 — Supporting File 1: advs75479‐sup‐0001‐SuppMat.docx. [file ADVS-13-e75479-s002.docx]

Supporting Information

**Table S1**

**
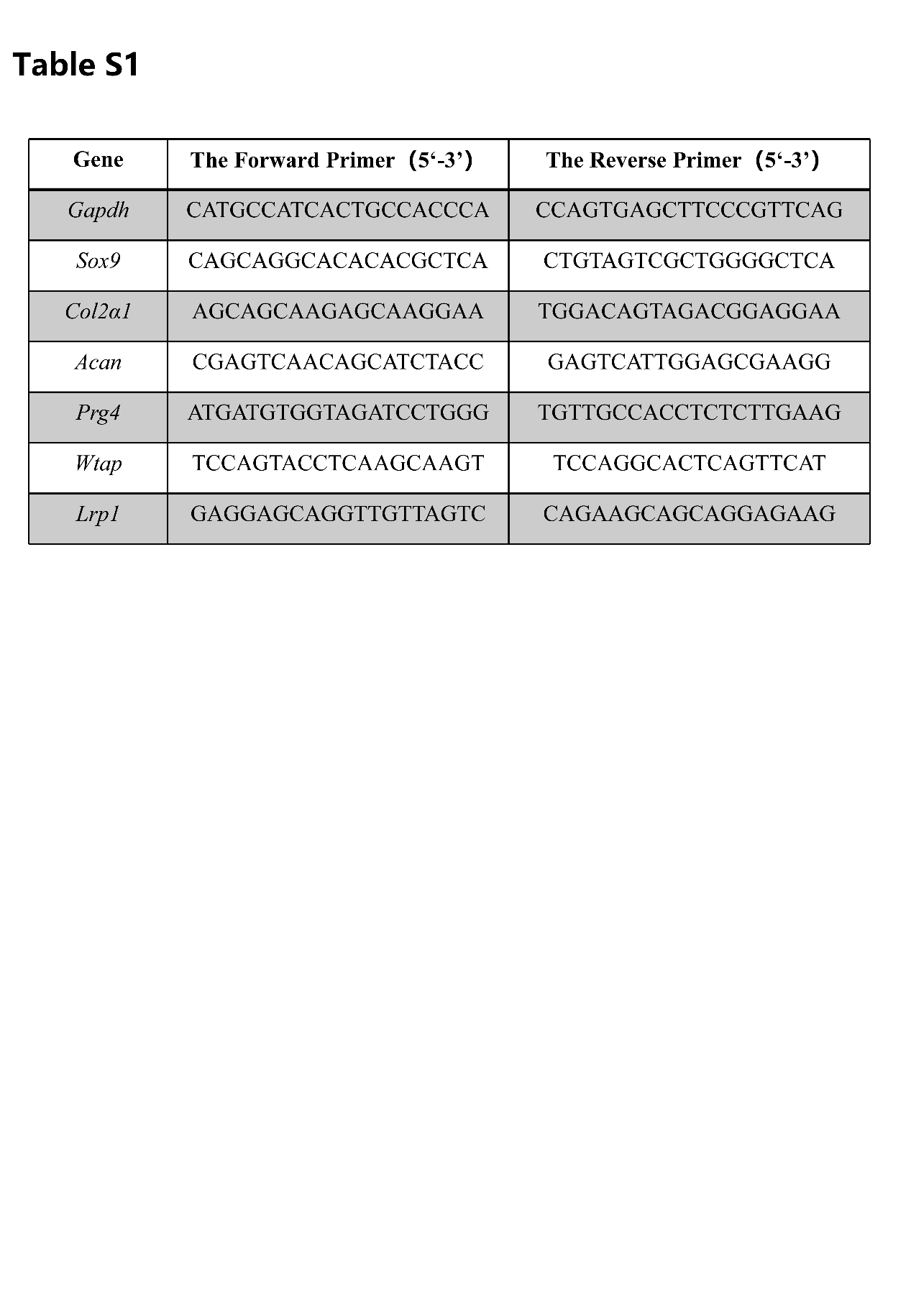
**

**Table S1.** The design sequences of all gene primers involved in the RT-qPCR of this study.

**Figure S1-S7**

**
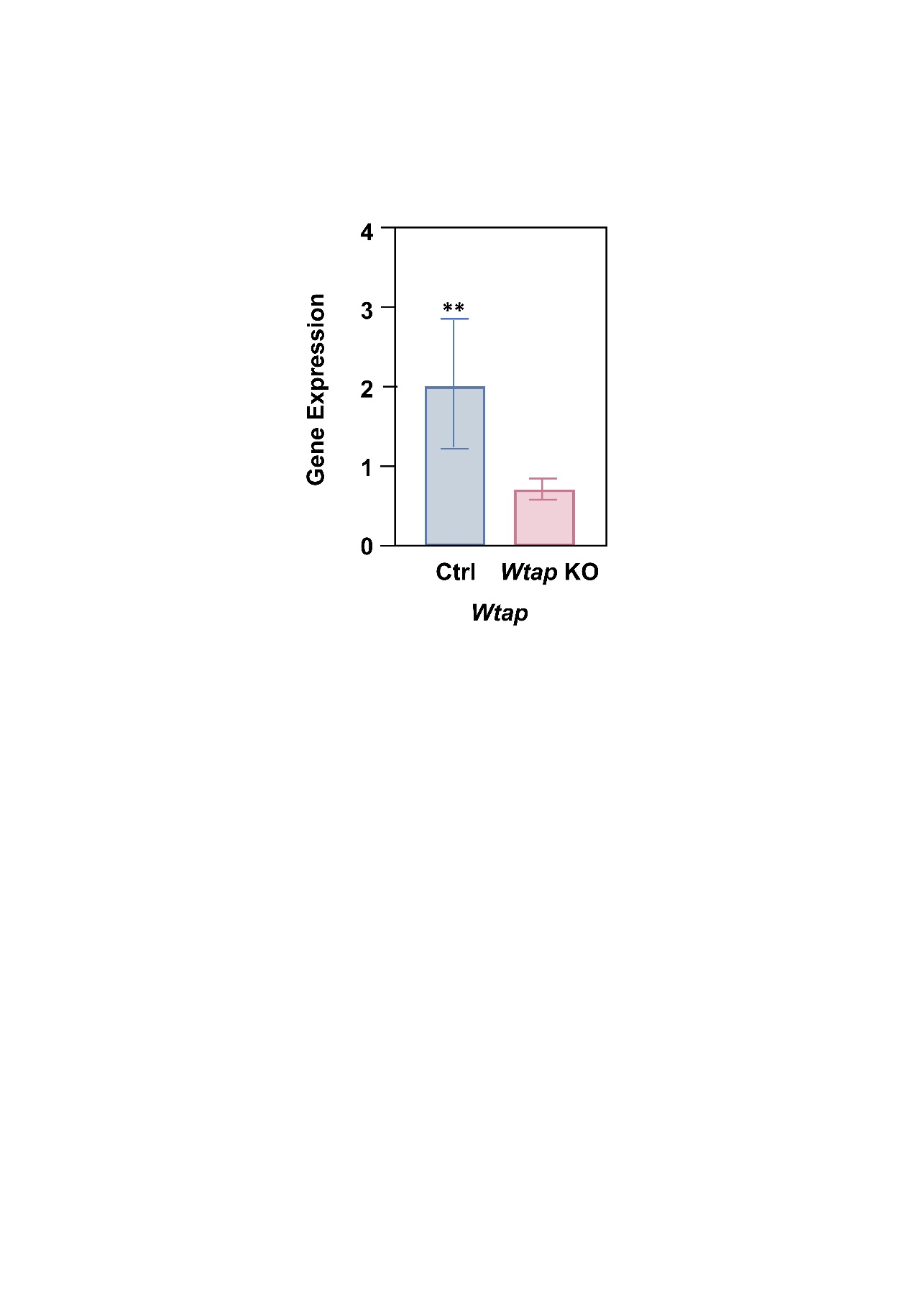
**

**Figure S1.** Efficient knockdown of *Wtap* in BMDMs *via* lentiviral transduction. RT-qPCR analysis of the expression of *Wtap* in BMDMs after *Wtap* knockout. Data are presented as means ± SD. ***p* < 0.01.


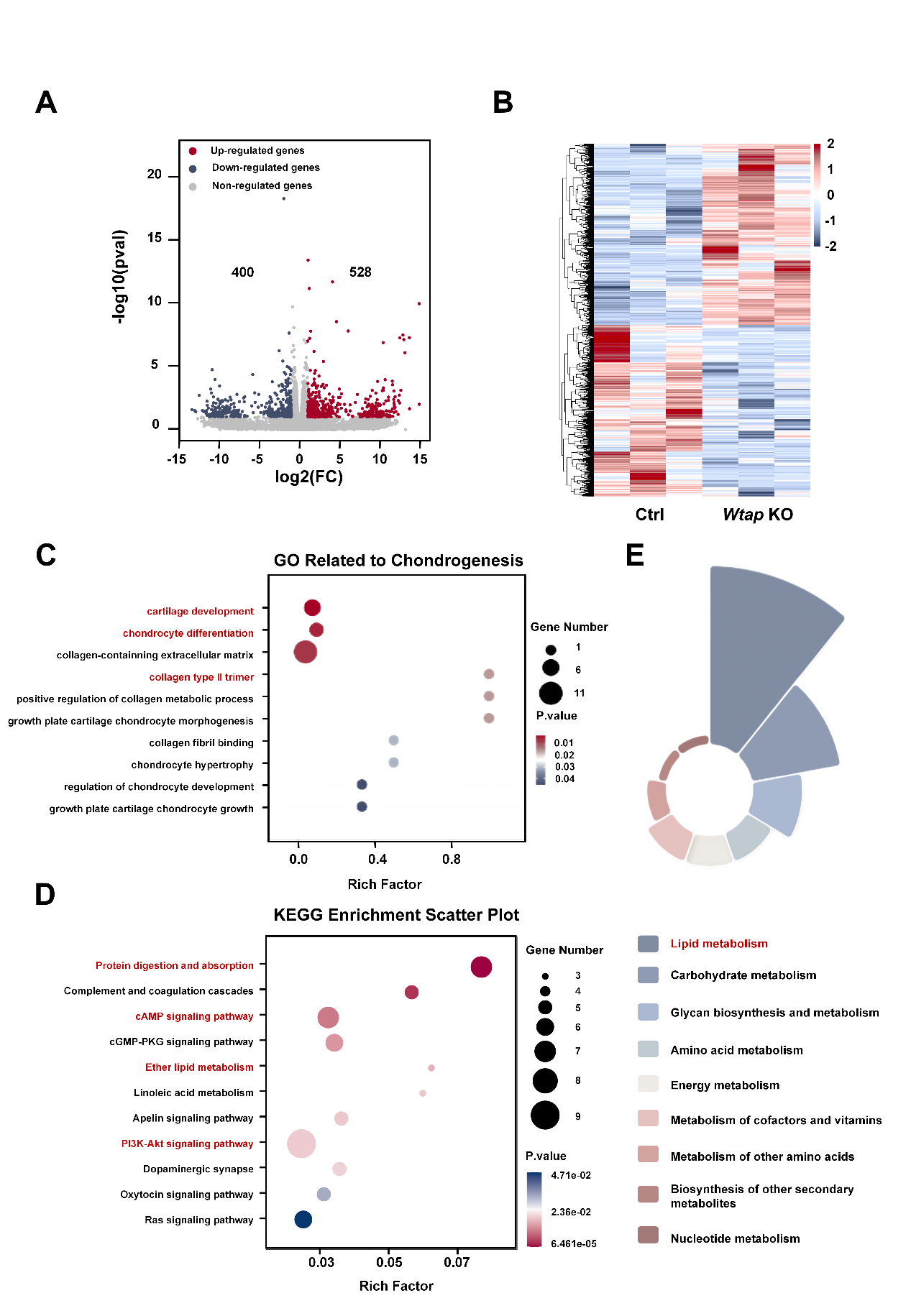


**Figure S2.** Transcriptomic analysis at 2 weeks reveals the role of lipid metabolism in WTAP-regulated cartilage regeneration. A) Volcano plot showing 528 upregulated and 400 downregulated DEGs between the *Wtap* KO and control groups. B) Heatmap of selected DEGs between the two groups. C) GO enrichment analysis analysis of the DEGs. D) KEGG enriched biological process and signaling pathway based on DEGs. E) KEGG enrichment analysis of metabolism-related DEGs following *Wtap* knockout.


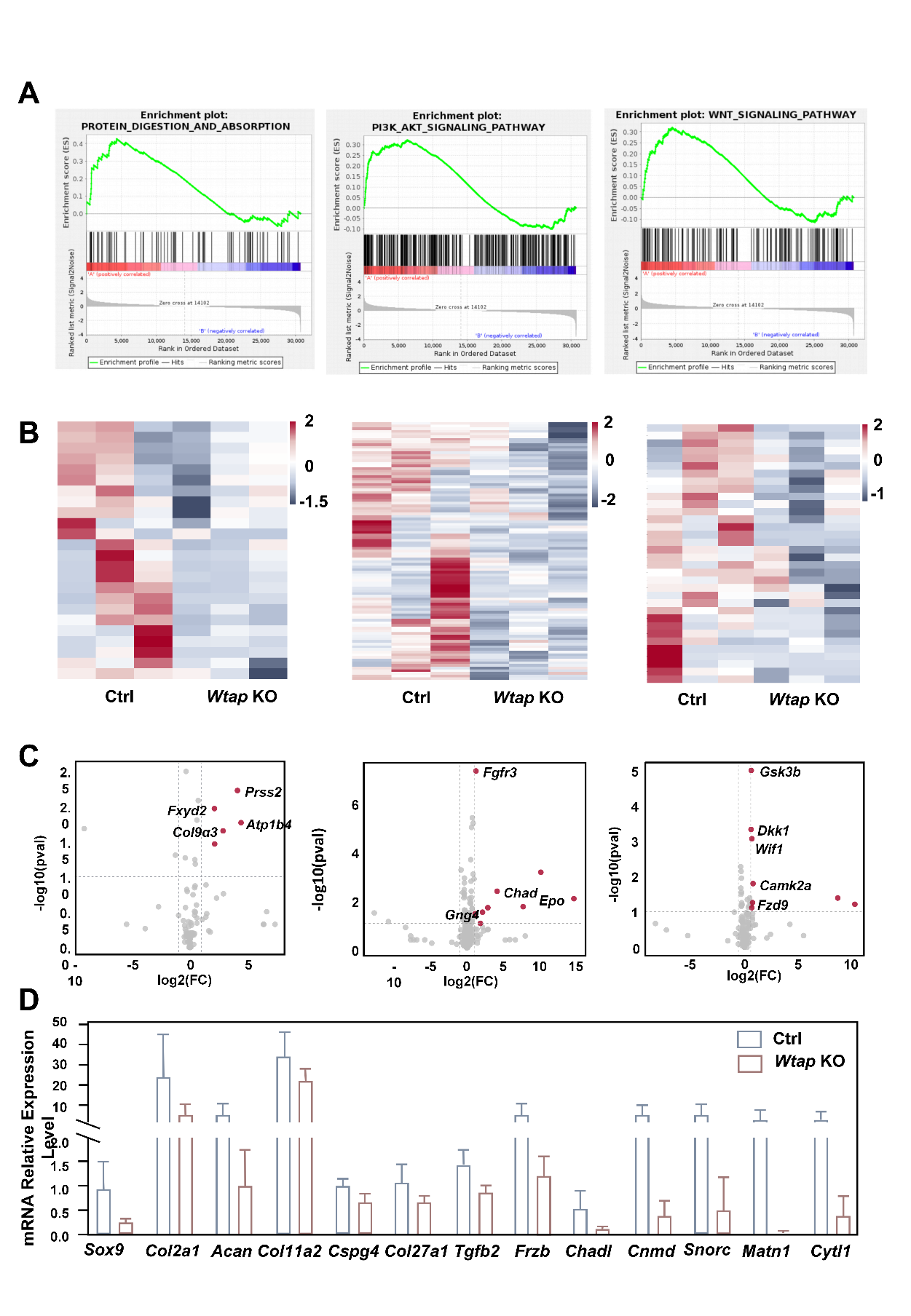


**Figure S3.** Transcriptomic analysis demonstrates that WTAP upregulates the expression of cartilage-related genes. A) GSEA of the Protein digestion and absorption, PI3K-Akt signaling pathway, and Wnt signaling pathway after *Wtap* knockout. B) Heatmap of DEGs in the three core pathways (from A) between the *Wtap* KO and control groups. C) Volcano plot of core genes from the three key pathways (from A) between the two groups. D) Relative expression analysis of chondrogenic differentiation-related genes in the *Wtap* KO group compared with the control.


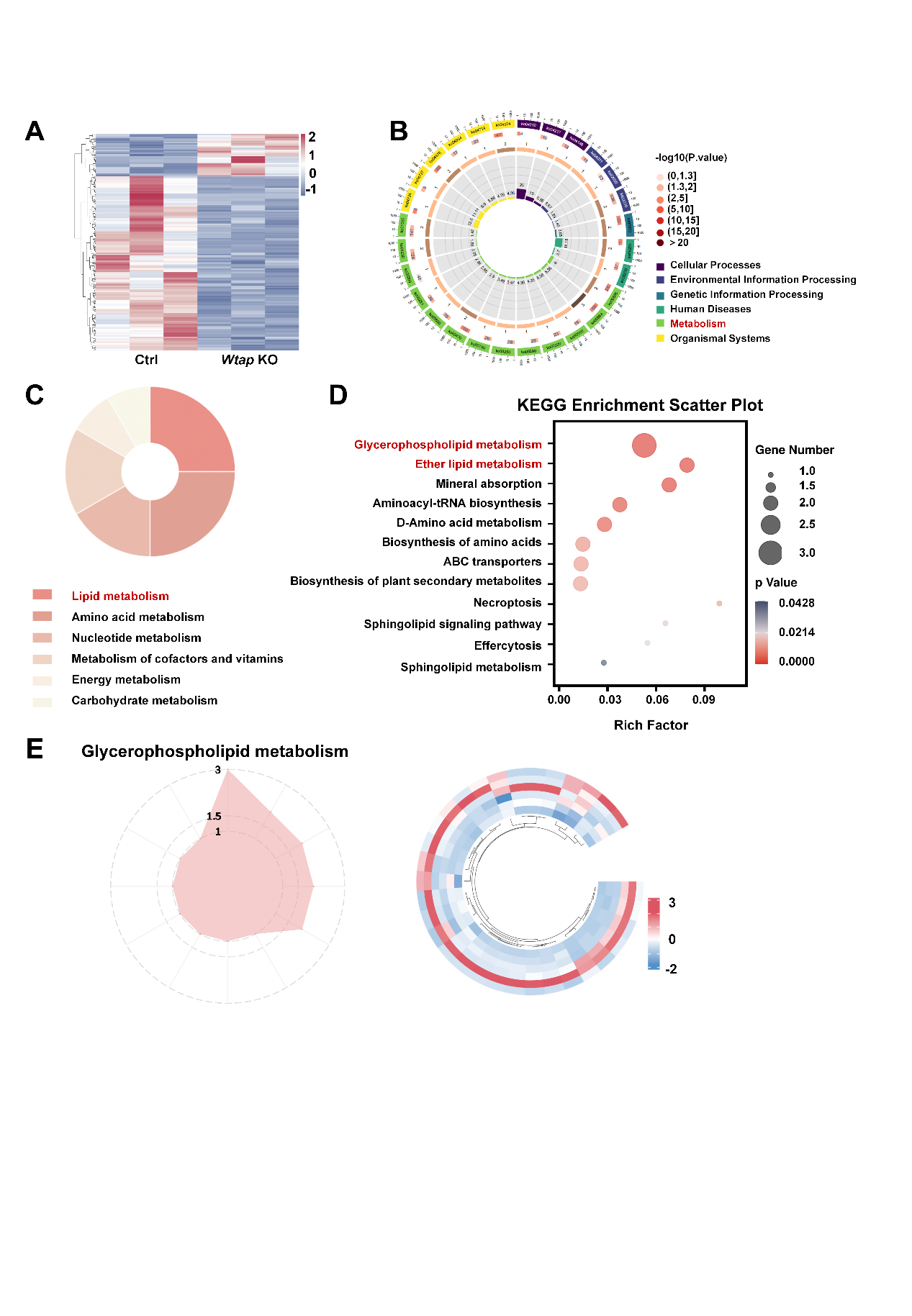


**Figure S4.** Metabolomic sequencing indicates the role of lipid metabolism in the WTAP-regulated cartilage regeneration process. A) Heatmap of DMs between the *Wtap* KO and control groups. KEGG enrichment analysis of the identified DMs (B), metabolism-related DMs (C), and lipid metabolism-related DMs (D) following *Wtap* knockout. E) Radar plot of KEGG-enriched lipid metabolism pathway (left) and the adjacent heatmap of DMs in lipid metabolism pathways (right), comparing the *Wtap* KO and control groups.


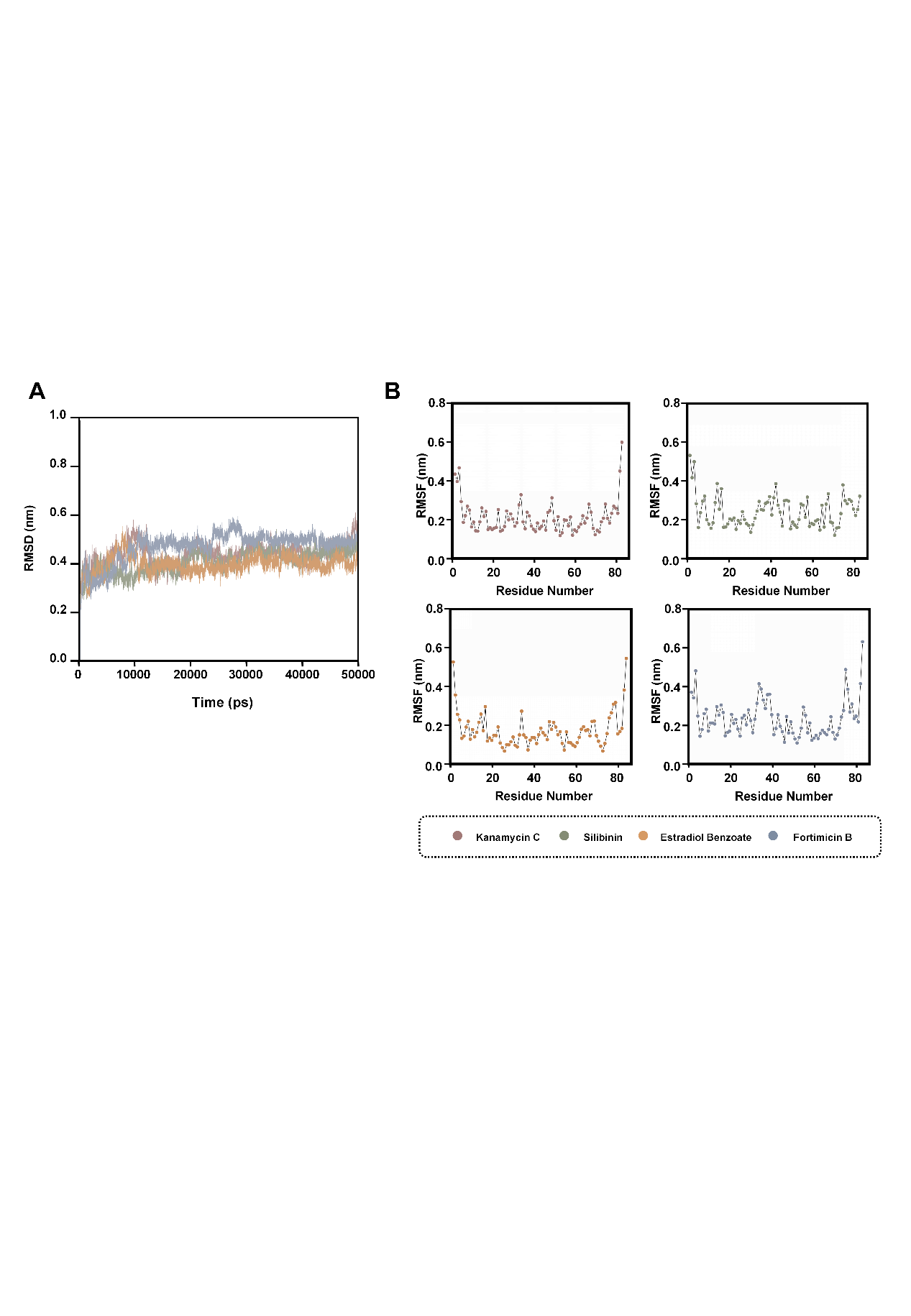


**Figure S5.** Silibinin and estradiol benzoate exhibit high binding affinity for the target LRP1. RMSD (A) and RMSF (B) analysis of LRP1 in complex with kanamycin C, silibinin, estradiol benzoate, and fortimicin B.


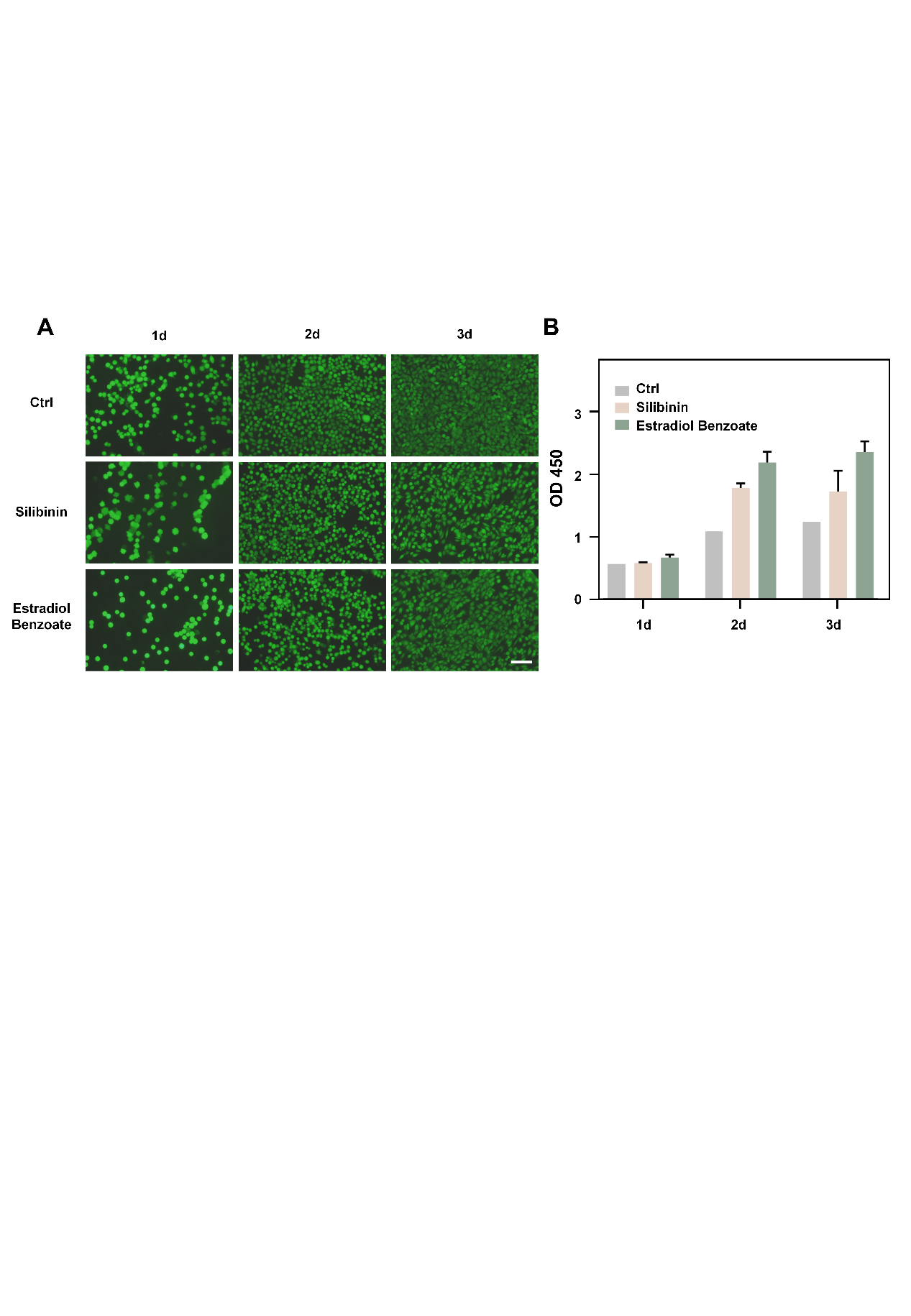


**Figure S6.** Silibinin and estradiol benzoate exhibit good biocompatibility. A) Respective image of Live/dead cell staining in BMDMs treated with silibinin and estradiol benzoate (scale bar: 100μm). B) CCK-8 results of BMDMs treated with silibinin and estradiol benzoat.

.


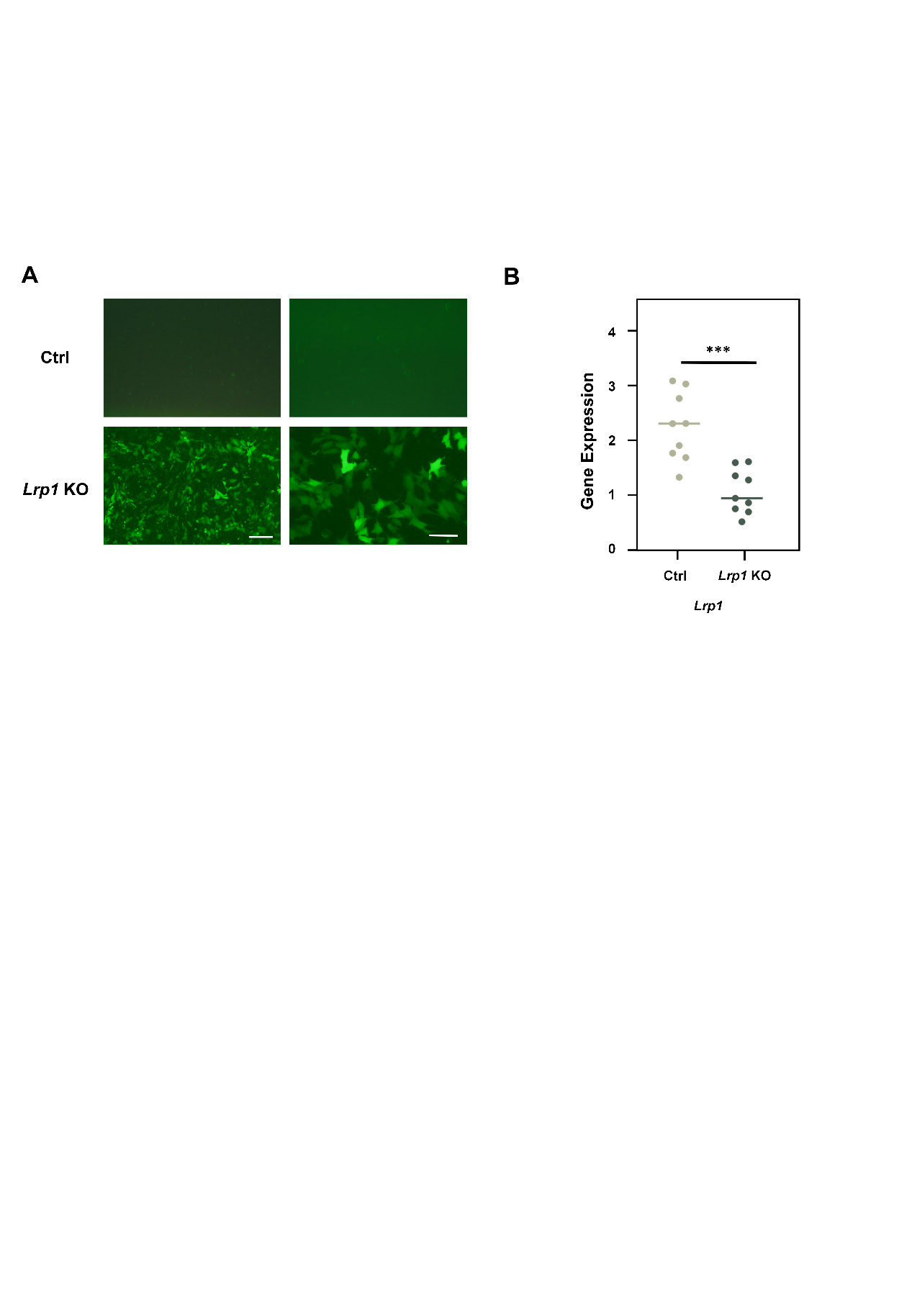


**Figure S7.** Generation of *Lrp1* KO BMDMs**.** A) Respective fluorescence images of the lentivirus-transfected efficiency between the *Lrp1* KO and control groups. Scale bar: 100 μm (left), scale bar: 50 μm (right). B) RT-qPCR analysis of gene expression of *Lrp1* in BMDMs after *Lrp1* knockout. Data are presented as means ± SD. ****p* < 0.001.


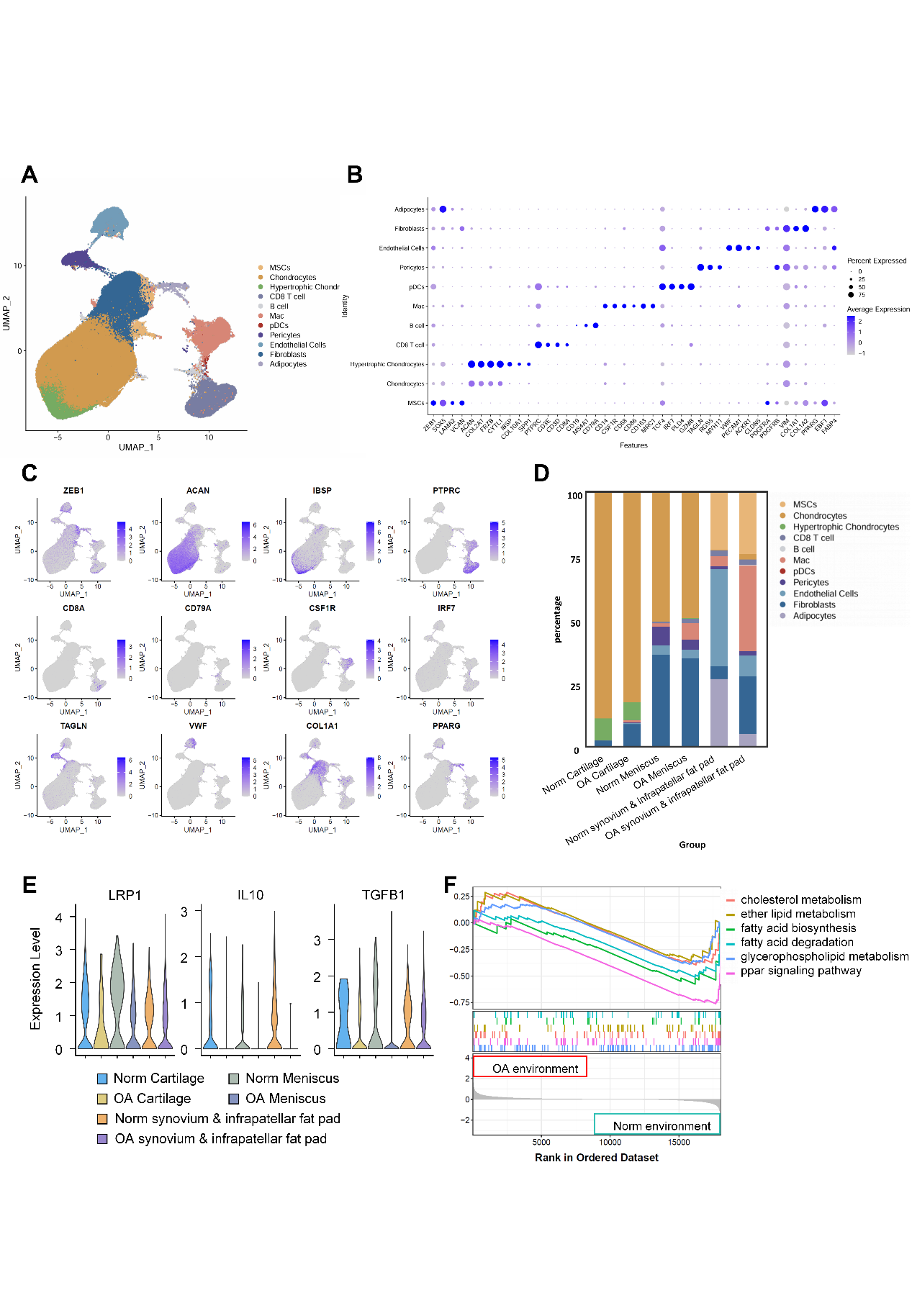


**Figure S8.** Human single-cell transcriptomic analysis reveals expansion of LRP1-low macrophages and suppression of lipid metabolic programs in osteoarthritic joint tissues. A) UMAP visualization of integrated single-cell RNA sequencing datasets from human normal and OA joint tissues, including cartilage, meniscus, synovium, and infrapatellar fat pad. Major cell populations were identified by unsupervised clustering. B) Dot plot showing canonical marker genes used to annotate major cell types, including MSCs, chondrocytes, hypertrophic chondrocytes, immune cells, endothelial cells, fibroblasts, and adipocytes. Dot size represents the percentage of expressing cells and color intensity represents average expression level. C) Feature plots illustrating representative marker gene expression across cell clusters, confirming accurate cell-type annotation. D) Relative cell-type composition across normal and OA tissues. Macrophages (Mac) are expanded in OA samples compared with corresponding normal tissues. E) Violin plots showing expression levels of LRP1 and downstream anti-inflammatory mediators (IL10 and TGFB1) across normal and OA joint tissues. LRP1, IL10, and TGFB1 are reduced in OA-associated macrophage-enriched environments. F) GSEA comparing OA and normal environments, demonstrating coordinated downregulation of lipid metabolism-related pathways in OA tissues, including cholesterol metabolism, fatty acid metabolism, glycerophospholipid metabolism, and PPAR signaling.
